# A convenient and practical synthesis of β-diketones bearing linear perfluorinated alkyl groups and a 2-thienyl moiety

**DOI:** 10.3762/bjoc.14.290

**Published:** 2018-12-27

**Authors:** Ilya V Taydakov, Yuliya M Kreshchenova, Ekaterina P Dolotova

**Affiliations:** 1 P.N. Lebedev Physical Institute of the Russian Academy of Sciences, Leninskiy prospect, 53, 119991 Moscow, Russian Federation; 2 Moscow Institute of Physics and Technology (State University). Institutskiy per., 9, 141701, Dolgoprudny, Moscow Region, Russian Federation; 3 D. Mendeleev University of Chemical Technology of Russia, Miusskaya sq. 9, 125047 Moscow, Russian Federation

**Keywords:** Claisen condensation, copper chelate, β-diketones, perfluorinated esters, thiophene

## Abstract

A versatile and robust synthetic protocol for the preparation of β-diketones bearing 2-thienyl and perfluorinated alkyl radicals of different length or a methyl group was developed. This protocol is suitable for the preparation of multigram quantities of diketones without cumbersome purification procedures. Moreover, the known method for purification of diketones via copper chelates was improved considerably.

## Introduction

Classical β-diketones have been studied for more than a century, and no doubt, they are the most popular O,O-ligands in the coordination chemistry of d- and f-elements [1–3]. These compounds are widely used as extractants in solvent–solvent extraction processes [4], in preparation of volatile complexes for chemical vapour deposition (CVD) technique [5–6], in syntheses of luminescent compounds [7], as starting materials in organic and heterocyclic chemistry [8–9] and in many other practical applications [10–11]. Diketones bearing perfluorinated radicals are very important for the design of highly effective luminescent materials based on lanthanide coordination compounds. Substitution of aliphatic radicals by perfluorinated ones in the molecules of ligands led to a significant increase in luminescence intensity due to suppression of multiphonon non-radiative relaxation [7,12–13].

**Scheme 1:**
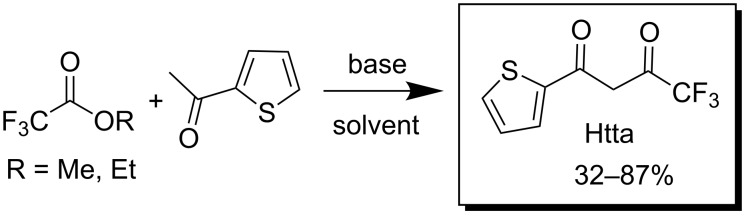
Synthesis of Htta.

Among all β-diketones, one derivative of thiophene, namely 2-thenoyltrifluoroacetone (Htta, 4,4,4-trifluoro-1-(2-thienyl)butane-1,3-dione), is produced on industrial scale and extensively used in the nuclear fuel separation cycle [14]. This compound was first obtained by Reid and Calvin in 1950 [15] by Claisen condensation of 2-acetylthiophene and ethyl trifluoroacetate in the presence of NaOEt in Et_2_O (Scheme 1). The purification procedure was laborious and included copper chelate precipitation and its subsequent acid decomposition. Afterwards, this synthetic protocol was substantially improved by other researchers. А number of different combination of solvents, bases and reaction conditions were described in literature for this condensation. For example, *t*-BuOK in benzene [16], NaOMe in methanol [17], NaH in Et_2_O [18], NaH in THF [19], NaOMe in Et_2_O [20] or LiHMDS in THF [21] were tested. The yields varied from 32 to 87%.

**Figure 1:**
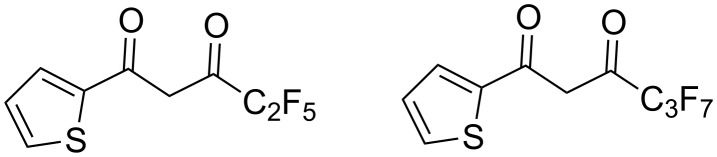
Known thiophene-based perfluorinated β-diketones.

Other β-diketones bearing a 2-thienyl moiety have been studied much less thoroughly. To our surprise, only two β-diketones bearing C_2_F_5_ [22] and C_3_F_7_ [23] groups were reported up to date in the literature (Figure 1). To the best of our knowledge, β-diketones with heavier linear perfluorinated radicals (C_4_–C_8_) are unknown.

During the ongoing project, we needed to synthesize a family of 2-thienyl diketones with various lengths of a perfluorinated side chain. Here we intend to report a versatile and robust practical method for the preparation of new β-diketones bearing 2-thienyl and various linear perfluоrinated (C_2_–C_8_) substituents. The method is also suitable for synthesizing 1-(2-thienyl)butane-1,3-dione and its analogues.

## Results and Discussion

A comprehensive analysis of the known methods revealed that sodium alkoxides (predominantly NaOMe or NaOEt) and Et_2_O were used most frequently as the base and solvent, respectively, in the Claisen condensation of 2-acetylthiophene (**1**) with various esters of aliphatic, aromatic or heterocyclic carboxylic acids [24] (Table 1). In our initial experiments we tested several commercial batches of NaOMe or NaOEt, along with samples freshly prepared in the laboratory.

**Table 1:** Optimization of reaction conditions.

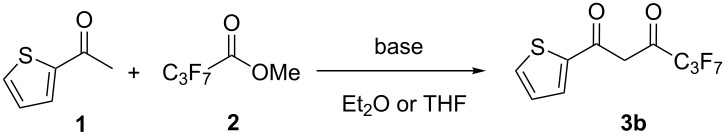						

entry	base	equiv of base	temp., °C^a^	variation^b^	solvent	yield, %^c^

1	NaOEt^d^	1	5/reflux	C	Et_2_O	78
2	NaOEt^d^	2.0	5/reflux	C	Et_2_O	91
3	NaOEt^d^	2.5	5/reflux	C	Et_2_O	92
4	NaOEt^e^	2	5/reflux	C	Et_2_O	63
5	NaOEt^f^	2	5/reflux	C	Et_2_O	57
6	NaOEt^g^	2	5/reflux	C	Et_2_O	58
7	NaOEt^h^	2	5/reflux	C	Et_2_O	91
8	NaOMe^d^	2	5/reflux	C	Et_2_O	93
9	NaOMe^d^	2	5/rt	C	Et_2_O	89
10	NaOMe^g^	2	5/rt	C	Et_2_O	61
11	NaOMe^h^	2	5/rt	C	Et_2_O	88
12	NaOEt^d^	2	5/reflux	A	Et_2_O	56
13	NaOEt^d^	2	5/reflux	B	Et_2_O	91
14	NaOEt^d^	2	5/reflux	C	THF	69
15	NaH^i^	2	5/reflux	C	Et_2_O	86
16	NaH^i^	1	5/reflux	C	THF	56
17	NaH^i^	2	5/reflux	C	THF	79
18	NaH^i^	2	5/rt	C	THF	90
19	NaH^j^	2	5/rt	C	THF	91

^a^Reaction temperature/temperature after addition; ^b^order of reagent mixing; for details, see text; ^c^estimated by GC; ^d^commercial, freshly opened container; ^e^commercial, after storage for 6 months; ^f^commercial, after storage for 14 months; ^g^freshly prepared, undried; ^h^freshly prepared, dried; ^i^commercial, 60% dispersion in oil; ^j^oil-free NaH.

In all the experiments, anhydrous Et_2_O was used as the solvent and the initial concentration of carbonyl compounds was 0.2 mol/L. Methyl heptafluorobutanoate (**2**) was chosen as the ester because the corresponding β-diketone **3b** is known and the staring material is readily available. All the experiments were performed using about 5 mmol of compounds. The ratio of reagents was 1:1 (ketone to ester), while from 1 up to 2.5 equivalents of a base were applied. The order of reagent mixing is also significant. It was found that mixing 2-acetylthiophene with a base in the absence of the ester (var. A) should be avoided due to notable darkening and self-condensation of the ketone, despite Prabhu’s recommendations [25]. Similar yields of a β-diketone were obtained if the ester of perfluorocarboxylic acid was first added to a base (var. B) or a mixture of the ester and ketone was introduced dropwise to a suspension of a base (var. C). The latter mode is preferable since the reaction can be controlled easily. The reaction temperature should be maintained below +5 °C during the addition. To complete the reaction, short refluxing (2–3 h) or stirring of the reaction mixture at room temperature overnight is required.

Highly active alkoxides were prepared in the laboratory by dissolution of sodium metal in excess anhydrous alcohol (MeOH or EtOH) followed by evaporation of the resulting solution under reduced pressure (rotary evaporator, 60 °C, 5 Torr). The residue was pulverized in dry Ar atmosphere and dried at 0.05 Torr and 60 °C for at least 5 h.

The quality of the alkoxide dramatically affects the yields of a β-diketone, while the stoichiometry is much less significant. The best results were achieved with commercial NaOMe or NaOEt from freshly opened metal cans, while old samples were much less reactive. We believe that slow degradation of alkoxides take place during storage under ambient conditions. Nevertheless, these samples of alkoxides are still suitable for many reactions other than the Claisen condensation of 2-acetylthiophene.

The problem of degradation of sodium alkoxides upon storage and determination of their activity as basic catalysts is quite complex. A few analytical methods were developed to measure the concentrations of NaOR and NaOH. They include the potentiometric titration with modified Karl Fisher’s reagents [26], spectrophotometric determination of NaOR with α-santonin [27], thermometric determination of alkoxides [28] and some other special techniques. Unfortunately, these methods are too laborious for a common synthetic laboratory. The effect of sodium ethoxide quality on the yields of a fluorinated β-diketone was mentioned in the pioneering works of Henne at al. concerning the Claisen condensation of perfluorinated esters [29].

Since preparation of active alkoxides is cumbersome and the yields are unstable with commercial reagents, NaH was tested as an alternative base. The preliminary results were comparable with those obtained with the most active samples of NaOMe or NaOEt. Optimization of the reaction conditions was made in order to achieve better and stable yields of β-diketones.

Diethyl ether is not the solvent of choice for this reaction due to the low boiling point, high volatility, and low solubility of sodium salts of diketones in this solvent. As far as NaH is also insoluble in organic solvents, the reaction medium is heterogeneous all the time, and strong effervescence makes the reaction unsafe and difficult to control. We revealed that THF is a much more suitable solvent. Additional advantages of THF include its high miscibility with both 2-acetylthiophene and even long-chain perfluorinated esters, and the high solubility of sodium enolates in it. This fact makes it possible to obtain homogeneous reaction mixtures after completion of condensation. The optimum molar ratio of ketone, ester and NaH was found to be 1:1:2. The excess NaH acts as an alcohol and water scavenger, thus making the synthetic protocol more robust. Commercial NaH is supplied in a relatively safe form of 60% dispersion in mineral oil. Although other researchers recommended to use this dispersion in native form [18–19,25], we have found that mineral oil should be removed before the synthesis. The results of the optimization experiments are summarized in Table 1.

The safest way to remove oil is by washing sodium hydride by means of decantation directly in the reaction flask. This procedure must be conducted under dry argon blanket and NaH must be maintained permanently wetted with anhydrous solvents.

Washing with two portions of hexane (each approx. 50 mL for a 4 g portion of the suspension) and with one portion of THF is usually sufficient. Washing does not affect the yields or the course of the reaction but significantly facilitates the purification process.

The best results were obtained if THF solution of carbonyl compounds was slowly added dropwise to a suspension of NaH in THF, while the temperature was maintained below +5 °C. Upon completion of the condensation, the reaction mixture should be kept at room temperature for 5–10 h, because prolonged reflux, as recommended by other researchers [19,25], notably decreases the yields and purity of β-diketones.

Condensation with NaH has an induction period, and after some time it can become dangerously vigorous due to the autocatalytic nature of the reaction. Since the alkoxide that is formed during the reaction acts as a catalyst [30], addition of small amounts (200–500 μL) of anhydrous EtOH (in a reaction using 100 mmol of compounds) to the initial NaH suspension in THF makes the reaction fully controllable. As it was mentioned before, an excess of NaH was used to improve the yields of diketones. It must be decomposed by careful addition of an anhydrous alcohol (MeOH or EtOH) before treatment of the reaction mixture with an aqueous acid. With this precaution, the whole synthetic procedure is safe. The results of the experiments are presented in Table 2.

**Table 2:** Preparation of perfluorinated β-diketones.

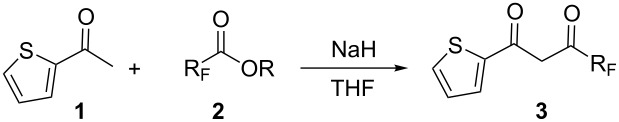			

compound	R_F_	R	yield, %^a^

**3a**	C_2_F_5_	Et	52 (92)
**3b**	C_3_F_7_	Me	66 (93)
**3c**	C_4_F_9_	Et	71 (82)
**3d**	C_5_F_11_	Me	75 (88)
**3e**	C_6_F_13_	Me	76 (83)
**3f**	C_7_F_15_	Et	77^b^
**3g**	C_8_F_17_	Me	83^b^

^a^Isolated by direct distillation and the total isolated yield (in parentheses); the total isolated yield means that an additional amount of a diketone was isolated via copper chelate from the low boiling fractions after distillation; ^b^isolated by distillation, no additional diketone was isolated as a copper chelate.

**Scheme 2:**
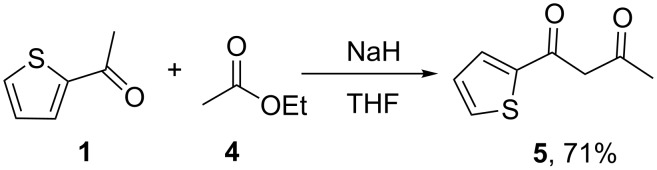
Preparation of 1-(2-thienyl)butane-1,3-dione (**5**).

Preparation of aliphatic diketones by the same method is much more difficult due to self-condensation of esters. We tested the procedure suggested for the preparation of 1-(2-thienyl)butane-1,3-dione (**5**) by condensation of 2-acetylthiophene with ethyl acetate (Scheme 2).

A large excess of EtOAc was used to facilitate cross-condensation, but formation of acetylacetone as a side product was always observed. It was found that a 60% dispersion and washed NaH gave comparable yields of impure diketone, and additional purification was needed in all the cases. Mineral oil can be removed from the product by vacuum distillation, because the diketone is a relatively low boiling compound. However, after the two-step purification procedure, the yield of pure product was about 71%, that is comparable with the other methods described in literature [31–32]. No additional optimization of reaction conditions was made for aliphatic esters.

Since the Claisen condensation is accompanied by formation of side products, purification of a diketone is usually difficult. Steam distillation of the reaction mixture followed by precipitation of metal (Cu, Mg, Mn, Fe) chelates and their acidic decomposition [22–23] or preparative chromatography [33–34] were used to obtain a pure product. We have found that in most cases, simple vacuum distillation of the crude reaction mixture is sufficient to obtain pure β-diketones with fluorinated side-chain. Nevertheless, precipitation of the Cu chelate is mandatory for purification of 1-(2-thienyl)butane-1,3-dione (**5**) and very useful for isolation of short-chain fluorinated diketones **3a**–**e** from low boiling fractions after distillation.

Copper salts of diketones **6** were prepared by a simplified method: the crude reaction mixture was added in a small portion to a vigorously stirred hot solution of Cu(OAc)_2_ (approx. 17 g per 100 mL of water) in 0.1% aqueous AcOH, the resulting suspension was cooled to room temperature, and the salt was separated by filtration and subsequently washed with water and hexane. The air-dried product is pure enough for the isolation of the β-diketones.

**Scheme 3:**
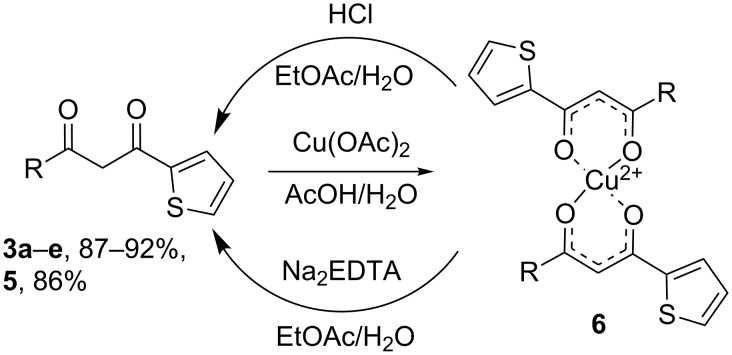
Preparation and cleavage of copper chelates of β-diketones.

Several methods for decomposition of copper chelates were described in literature. Usually sulfuric acid with various concentrations (10–95%) was applied [22–23,34–35], but the thiophene ring is known to be sensitive to sulfuric acid. For this reason, we developed two new mild protocols for the regeneration of diketones (Scheme 3). A vigorously stirred or shaken two-phase mixture of EtOAc and an aqueous solution of the reagent were used, and a finely ground solid Cu chelate was added to it in small portions.

The choice of the reagent depends on the p*K*_a_ of the diketone: a saturated solution of disodium EDTA (Trilon B) was found to be most effective for less acidic aliphatic compounds, while 10% HCl was applied for much more acidic fluorinated ones. Since the solubility of Trilon B in water is relatively low (10 g/100 mL of H_2_O), several successive extractions can be required to decompose the whole amount of a chelate. Upon completion of decomposition, the organic phase was separated, the solvent was evaporated and the pure diketone was isolated in analytically pure form by distillation. No additional laborious purification was needed.

**Scheme 4:**
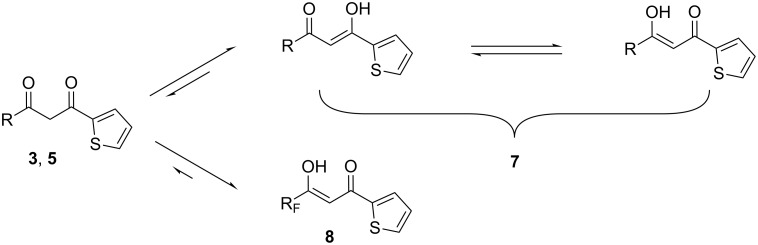
Keto-enol equilibrium of β-diketones.

The purity and composition of all the diketones synthesized were confirmed by a combination of methods, including NMR spectroscopy. It is known that β-diketones exist in solution as a mixture of keto and enol forms. This tautomeric equilibrium strongly depends on a variety of factors. Of these, the nature of the diketone and the solvent are most important [30]. We have found that in CDCl_3_ solutions, all the new diketones bearing fluorinated groups exist solely in one enol form **8**. It was confirmed by ^1^H NMR spectra: a broad signal of the OH group (around 14.9–15.3 ppm) along with a sharp signal of protons attached to the C=C enol bond (around 6.5 ppm) were observed in all spectra. Conversely, no signals of the CH_2_ moiety were observed [36]. As one might have expected, a solution of 1-(2-thienyl)butane-1,3-dione (**5**) in CDCl_3_ contains both forms, keto (**5**) and enol (**7**), in a molar ratio of 1:5 (Scheme 4).

## Conclusion

In summary, a versatile and robust synthetic protocol for the preparation of β-diketones bearing 2-thienyl and perfluorinated alkyl radicals with various lengths or a methyl group was developed. This protocol is suitable for the preparation of multigram quantities of diketones without cumbersome purification procedures. A number of new diketones were synthesized and fully characterized. In addition, the known purification method for diketones via copper chelates was improved substantially.

## Supporting Information

File 1 Experimental procedures and characterization data for compounds **3a–g** and **5**.

File 2 Copies of ^19^F and ^13^C NMR spectra and LR mass spectra of compounds **3a–g** and **5**.
