# Supplementary material for: A convenient and practical synthesis of β-diketones bearing linear perfluorinated alkyl groups and a 2-thienyl moiety
Source: Beilstein J Org Chem. 2018 Dec 27;14:3106–11. doi: 10.3762/bjoc.14.290 (PMC6317410; doi:10.3762/bjoc.14.290)
Supplement: File 1 — Experimental procedures and characterization data for compounds 3a–g and 5. [file Beilstein_J_Org_Chem-14-3106-s001.pdf]

BEILSTEIN JOURNAL OF ORGANIC CHEMISTRY

Supporting Information

for

A convenient and practical synthesis of β-diketones bearing linear perfluorinated alkyl groups and a 2-thienyl moiety

Ilya V. Taydakov, Yuliya M. Kreshchenova and Ekaterina P. Dolotova

Beilstein J. Org. Chem. **2018,** *14,* 3106–3111. doi:10.3762/bjoc.14.290

Experimental procedures and characterization data for compounds 3a–g and 5

License and Terms: This is a supporting information file under the terms of the Creative Commons Attribution License (http://creativecommons.org/licenses/by/4.0). Please note that the reuse, redistribution and reproduction in particular requires that the authors and source are credited.
The license is subject to the *Beilstein Journal of Organic Chemistry* terms and conditions: (https://www.beilstein-journals.org/bjoc)

Table of contents:

General information:	S2
General procedures for the preparation of **3a–g**:	S3
Preparation of compound **5:**	S4
General methods for the preparation and cleavage of copper chelates:	S5
Characterization data (**3a–g**; **5**):	S5
References:	S12

General information

2-Acetylthiophene, sodium hydride (60% dispersion in mineral oil) and other common chemicals were purchased from Acros Organics (Belgium). Ethyl or methyl esters of perfluorinated carboxylic acids were purchased from SIA "P&M-Invest" Ltd. (Moscow, Russia). Diethyl ether or THF were distilled over sodium metal/benzophenone under Ar atmosphere just before use. Ethyl acetate (for condensation) and esters of perfluorinated carboxylic acids were kept over 4 Å molecular sieves. Oven-dried glassware was used in the condensation step and manipulations were conducted under dry Ar blanket.

Caution! Dry or oil-free sodium hydride is highly pyrophoric and reacts violently with water. It should never be exposed to air during the washing procedure. It must be wetted by a solvent all the time and an inert atmosphere must be maintained during the manipulations. Traces of NaH in hexane after the washing procedure must be decomposed by careful addition of anhydrous ethanol followed by 96% ethanol before collecting it in a waste container.

NMR spectra were recorded at ambient temperature on a Bruker AM-300 instrument operating at 300, 282.5 and 75.5 MHz for ^{1}H, ^{19}F and ^{13}C nuclei respectively. Chemical shifts were referenced to $CHCl_3$ (7.26 ppm), $CDCl_3$ (77.2 ppm) and $CFCl_3$ (0.00 ppm) for ^{1}H, ^{13}C and ^{19}F spectra, respectively. ^{13}C NMR spectra were also recorded on a Bruker DXR-500 and AV-600 instruments operated at 125.8 and MHz respectively. FTIR spectra were obtained on a Perkin-Elmer system Spectrum One 100 FTIR spectrometer in KBr pellets. GC–MS analysis and LRMS were performed on a Thermo DSQ II/Thermo Trace instrument; the ionization energy was 70 eV. HRMS was performed on a Bruker MicroTOF instrument with an ESI ionization source in the positive ion detection mode. Melting points were measured on a Büchi Melting Point M-560 apparatus and are uncorrected. Elemental microanalysis was performed in the

Laboratory of microanalysis of Nesmeyanov Institute of Organoelement Compounds, Moscow, Russia.

^{13}C-NMR spectra for fluorinated diketones are considerably less informative and more difficult to acquire and to interpret. Due to numerous C–F spin-spin interactions, the lines in a spectrum are repeatedly splitted and their intensities are very low, especially for quaternary carbon atoms in long-chain radicals. Out attempts to improve the quality of ^{13}C-NMR spectra by utilizing high-field NMR spectrometers (Bruker DXR-500 and AV-600) also failed. With very long acquisition time, spectra became too complicated due to numerous signals of minor products and impurities, while with short acquisition times, only signals of carbon atoms that are not connected to fluorine atoms were recorded. The next problem is the high multiplicity of the signals – for long-chain fluorinated substituents it was impossible to resolve all multiplets even on the 600 MHz instrument. Therefore, these spectra are not fully described in the experimental section.

General method for the preparation of fluorinated β-diketones (3a–g)

Sodium hydride (4.0 g, 100 mmol, 60% dispersion in mineral oil) was placed in a 500 mL round-bottom flack and 50 mL of dry hexane was added. The suspension was stirred for a short time with a magnetic stirring bar and kept until the hexane layer became clear (20–30 min). Hexane was cautiously removed with a glass pipette until a wet paste of NaH remained. This procedure was repeated twice. After that, THF (150 mL) was added with vigorous stirring. Anhydrous EtOH (0.5 mL) was added in one portion at 0 °C, followed by the dropwise addition of a solution of 2-acetylthiophene (6.3 g, 50 mmol, 5.42 mL) and methyl or ethyl ester of the respective perfluorinated carboxylic acid (50.5 mmol) in 30 mL of THF. The white suspension rapidly turns pink and then reddish-brown. Caution ! Hydrogen evolved during the addition and notable effervescence was observed. The rate of addition was maintained to control the evolution of hydrogen; usually the addition takes 60–80 min. When gas evolution

ceased, the cooling bath was removed and the reaction mixture was stirred for 5 h. After that, the dark brown solution with a small amount of precipitate was re-cooled to 0 °C, 10 mL of anhydrous EtOH was added slowly to decompose traces of NaH, and the resulting solution was stirred for 30 min. The solvent was removed by evaporation under reduced pressure (100 Torr, bath temperature 40 °C), then EtOAc (40 mL) and subsequently a mixture of conc. HCl (20 mL) and crushed ice (80 mL) were added to the residue. The resulting turbid liquid was shaken by hand until two clear layers formed. The organic phase was separated and the aqueous phase was then extracted with EtOAc (3 × 50 mL). The combined organic fractions were washed with brine (50 mL), dried over $MgSO_4$ and evaporated to dryness. The resulting brown oil was distilled in vacuo (2–3 Torr). The main fraction is pure enough. Additional portions of pure diketone can be separated from low-boiling fractions by precipitation as a copper salt. Decomposition should be performed with HCl (procedure A).

1-(2-Thienyl)butane-1,3-dione (5)

To a vigorously stirred suspension of NaH (2.4 g, 60 mmol, 60% dispersion in mineral oil) in 120 mL of THF, 0.2 mL of anhydrous EtOH was added in one portion. Stirring was continued and a mixture of 3.78 g (30 mmol) of 2-acetylthiophene and 36 mL (32.47 g, 368 mmol) of dry EtOAc was added dropwise at 0 °C. The resulting reddish-brown suspension was stirred at 0 °C for 5 h and left at a room temperature overnight. Caution ! Hydrogen evolved during the addition and notable effervescence was observed. The reaction mixture was cooled to 0 °C again, 10 mL of anhydrous EtOH was added slowly to decompose traces of NaH, and the resulting solution was stirred for 30 min. The solvent was removed by evaporation under reduced pressure (100 Torr, bath temperature 40 °C) and EtOAc (40 mL), then a mixture of conc. HCl (20 mL) and crushed ice (80 mL) were added to the residue. The resulting turbid liquid was shaken

by hand until two clear layers formed. The organic phase was separated and the aqueous phase was extracted with EtOAc (3 × 50 mL). The combined organic fractions were washed with brine (50 mL), dried over $MgSO_4$ and evaporated to dryness. The resulting brown oil was distilled and the fraction with b.p. 105–115 °C/3 Torr (5.2 g) was collected. According to NMR data, it was not pure enough, and it was purified via the copper chelate (procedure B).

Copper chelate preparation

The weight of $Cu(OAc)_2 \cdot H_2O$ was calculated assuming 100% purity of the diketone to be purified. To purify 100 mmol of a diketone, 40 g (200 mmol) of copper acetate monohydrate and 300 mL of 0.01 M aqueous AcOH were used. The salt was dissolved with stirring in the hot acid solution. The resulting dark greenish-blue solution was filtered hot through a loose cotton plug and the filtrate was re-heated to 90 °C. The diketone was added to the nearly boiling solution with vigorous mechanical stirring, after that the mixture was cooled to room temperature. The resulting precipitate was separated, washed thoroughly with warm 0.01 M aqueous AcOH, dried on a filter and washed several times with a sufficient amount of hexane. The copper chelate was dried in air to a constant weight.

Copper chelate decomposition by HCl (highly acidic diketones, procedure A)

A finely ground copper salt (50 mmol) was added in several portions with shaking to a two-phase mixture of EtOAc (50 mL) and 10% aqueous solution of HCl (50 mL) placed in a 250 mL separation funnel. The resulting mixture was vigorously shaken until all the chelate dissolved. The organic layer was separated, and the aqueous layer was extracted with EtOAc (3 × 80 mL). The combined organic fractions were washed successively with 20 mL of 10% HCl and 50 mL of brine, dried over $MgSO_4$ and evaporated to dryness. The diketone was purified by vacuum distillation.

Copper chelate decomposition by Trilon B (low acidic diketones, procedure B)

A finely ground copper salt (50 mmol) was added in several portions with shaking to a two-phase mixture of EtOAc (100 mL) and saturated aqueous solution of Trilon B (150 mL) placed in a 500 mL separation funnel. The resulting mixture was vigorously shaken. After the separation of layers, the blue aqueous layer was discarded. Extraction was continued with 50 mL portions of the Trilon B solution until it remained colorless (2–4 times). The organic fraction was washed with brine (50mL), dried over $MgSO_4$ and evaporated to dryness. The diketone was purified by vacuum distillation.

4,4,5,5,5-Pentafluoro-1-(thiophen-2-yl)pentane-1,3-dione (3a)

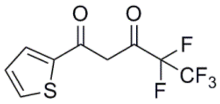

Colorless oil; yield: 7.0 g (52%) was obtained by direct distillation and 5.5 g was isolated from low-boiling fractions; yield: 12.5 g (92%); bp 77-78 °C (3 Torr) (lit. 95.5 °C /16 Torr) [1].

IR (KBr): 1639, 1592, 1411, 1330, 1203, 1069, 1003, 723cm^{-1}.

^{1}H NMR ($CDCl_3$, 300 MHz): δ = 14.99 (broad s, 1H, OH), 7.87 (d, 1H, J=3.7Hz CH), 7.77(d, 1H, J=4.8Hz,CH), 7.22 (t, 1H, J=4.5Hz, CH), 6.52 (s, 1H, CH=C).

^{19}F NMR ($CDCl_3$, 282.5 MHz): δ = -83.6 (s, 3F, CF_3), -124.57 (s, 2F,CF_2).

^{13}C NMR ($CDCl_3$, 75.5 MHz): δ = 182.61 (s, 1C, C=O), 172.70 (t, 1C, J=26.6Hz, OH-C=), 139.41(s,1C, C_{Th}), 135.47 (s,1C, C_{Th}), 132.93 (s,1C, C_{Th}),128.97 (s,1C, C_{Th}), 123.97, 120.17, 116.37, 111.75, 108.31, 104.7 (m, 2C, CF_3-CF_2), 95.01 (t, 1C, J=3.7Hz, CH=).

MS (EI, 70 eV): m/z (%) = 272 (45) $[M]^+$, 153(100) $[M-C_2F_5]^+$, 111(44) $[ThCO]^+$, 69(59) $[CF_3]^+$.

HRMS (ESI+): m/z $[M]^+$ calcd for $C_9H_5F_5O_2S$: 271.9930; found: 271.9934.

Anal. Calcd for $C_9H_5F_5O_2S$: C, 39.71; H, 1.85; F, 39.90. Found: C, 39.89; H, 1.73; F, 40.14.

4,4,5,5,6,6,6-Heptafluoro-1-(2-thienyl)hexane-1,3-dione (3b)

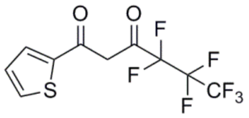

Yellow oil; yield: 10.7 g (66%) was obtained by direct distillation and 4.2 g was isolated from low-boiling fractions; yield: 14.9 g (93%); bp 87-88 °C (3 Torr) (lit. 99-102°C /5 Torr) [2].

IR (KBr): 1592, 1412, 1349, 1231, 1126, 1062, 872, 723 cm^{-1}.

^{1}H NMR ($CDCl_3$, 300 MHz): δ = 15.10 (broad s, 1H, OH), 7.88 (m, 1H, CH), 7.79(m, 1H, CH), 7.23 (m, 1H, CH), 6.50 (s, 1H, CH=C).

^{19}F NMR ($CDCl_3$, 282.5 MHz): δ = -81.40 (t, 3F, J= 8.3Hz, CF_3), -122.22 (d, 2F, J = 8.7Hz, CF_2), -127.63 (s, 2F, CF_2).

^{13}C NMR ($CDCl_3$, 125.8 MHz): δ = 182.01 (s, 1C, C=O), 172.97 (t, 1C, J=26.6Hz, OH-C=), 139.14(s,1C, C_{Th}), 135.32 (s,1C, C_{Th}), 132.78 (s,1C, C_{Th}),128.84 (s,1C, C_{Th}), 128.05; 120.91; 118.93; 116.64; 111.58; 109.50; 108.58; 107.42 (m, 2C, $C3F_7$) 95.05 (t, 1C, J=3.8Hz, CH=). Signals of CF_2 and CF_3 groups were not fully acquired and resolved.

MS (EI, 70 eV): m/z (%) = 322(18) $[M]^+$, 153(100) $[M-C_3F_7]^+$, 111(47) $[Th-CO]^+$, 69(63) $[CF_3]^+$.

HRMS (ESI+): m/z $[M]^+$ calcd for $C_{10}H_5F_7O_2S$: 321.9898; found: 321.9901.

Anal. Calcd for $C_{10}H_5F_7O_2S$: C,37.28; H, 1.56; F, 41.28. Found: C, 37.11; H, 1.59; F, 41.36.

4,4,5,5,6,6,7,7,7-Nonafluoro-1-(2-thienyl)heptane-1,3-dione (3c)

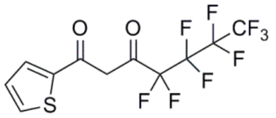

Yellow solid; yield: 13.2 g (71%) was obtained by direct distillation and 2.1 g was isolated from low-boiling fractions; yield: 15.3 g (82%); bp 90-92 °C (3 Torr); mp 28–29 °C.

IR (KBr): 1617, 1412, 1353, 1239, 1137, 866, 722 cm^{-1}.

^{1}H NMR ($CDCl_3$, 300 MHz): δ = 15.08 (broad s, 1H, OH), 7.87 (s, 1H, CH), 7.79(s, 1H, CH), 7.23 (m, 1H, CH), 6.50 (s, 1H, CH=C).

^{19}F NMR ($CDCl_3$, 282.5 MHz): δ = -81.68 (s, 3F, CF_3), -121.39 (t, 2F, J = 10.5Hz, CF_2), -124.06 (s, 2F, CF_2), -126.66 (s, 2F, CF_2).

^{13}C NMR ($CDCl_3$, 125.8 MHz): δ = 182.94 (s, 1C, C=O), 173.22 (t, 1C, J=26.6Hz, OH-C=), 139.10(s,1C, C_{Th}), 135.27 (s,1C, C_{Th}), 132.74 (s,1C, C_{Th}),128.83 (s,1C, C_{Th}),118.34; 116.12; 112.27; 109.93; 108.16 (m, 4C, C_4F_9) 95.01 (s, 1C, CH=). Signals of CF_2 and CF_3 groups were not fully acquired and resolved.

MS (EI, 70 eV): m/z (%) = 372(35) $[M]^+$, 153(100) $[M-C_4F_7]^+$, 111(35) $[ThCO]^+$, 69(33) $[CF_3]^+$.

HRMS (ESI+): m/z $[M]^+$ calcd for $C_{11}H_5F_9O_2S$: 371.9867; found: 371.9863.

Anal. Calcd for $C_{11}H_5F_9O_2S$: C,35.50; H, 1.35; F, 45.94. Found: C, 35.66; H, 1.31; F, 45.90.

4,4,5,5,6,6,7,7,8,8,8-Undecafluoro-1-(2-thienyl)octane-1,3-dione (3d)

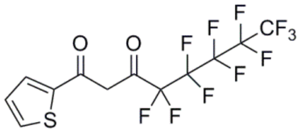

Light-yellow oil; yield: 15.9 g (75%) was obtained by direct distillation and 2.9 g was isolated from low-boiling fractions; yield: 18.8 g (88%); bp 92-95 °C (2 Torr).

IR (KBr): 1598; 1412; 1359; 1240; 1202; 1143; 863; 723 cm^{-1}.

^{1}H NMR ($CDCl_3$, 300 MHz): δ = 15.15 (broad s, 1H, OH), 7.88 (s, 1H, CH), 7.79(s, 1H, CH), 7.23 (m, 1H, CH), 6.51 (s, 1H, CH=C).

^{19}F NMR ($CDCl_3$, 282.5 MHz): δ = -81.61 (s, 3F, CF_3), -121.23 (s, 2F, CF_2), -123.31 (s, 4F, CF_2), -126.97 (s, 2F, CF_2).

^{13}C NMR ($CDCl_3$, 125.8 MHz): δ = 181.90 (s, 1C, C=O), 173.33 (t, 1C, J=26.7Hz, OH-C=), 139.10(s,1C, C_{Th}), 135.27 (s,1C, C_{Th}), 132.73 (s,1C, C_{Th}),128.83 (s,1C, C_{Th}), 118,31, 116.01, 112.49, 110.24, 108.17 (m, 5C, C_5F_{11}), 94.99 (s, 1C, CH=). The signals of CF_2 and CF_3 groups were not fully acquired and were not resolved.

MS (EI, 70 eV): m/z (%) = 422(28) $[M]^+$, 153(100) $[M-C_5F_{11}]^+$, 111(31) $[ThCO]^+$, 69(31) $[CF_3]^+$.

HRMS (ESI+): m/z $[M]^+$ calcd for $C_{12}H_5F_{11}O_2S$: 421.9835; found: 421.9838.

Anal. Calcd for $C_{12}H_5F_{11}O_2S$: C,34.14; H, 1.19; F, 49.50. Found: C, 34.02; H, 1.26; F, 49.31.

4,4,5,5,6,6,7,7,8,8,9,9,9-Tridecafluoro-1-(2-thienyl)nonane-1,3-dione (3e)

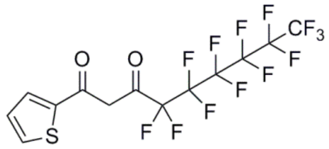

White solid; yield: 18.0 g (76%) was obtained by direct distillation and 1.6 g was isolated from low-boiling fractions; yield: 19.6 g (83%); bp 110-112 °C (2 Torr); mp 47–48 °C.

IR (KBr): 1591; 1411; 1237; 1209; 1147; 1071; 854; 723 cm^{-1}.

^{1}H NMR ($CDCl_3$, 300 MHz): δ = 14.85(broad s, 1H, OH), 7.88 (d, 1H, J=3.8Hz, CH), 7.79(d, 1H, J=4.8Hz, CH), 7.23 (t, 1H, J=4.5Hz, CH), 6.51 (s, 1H, CH=C).

^{19}F NMR ($CDCl_3$, 282.5 MHz): δ = -81.62 (s, 3F, CF_3), -121.15 (s, 2F, CF_2), -122.47 (s, 2F, CF_2), -123.13 (s, 2F, CF_2), -123.53 (s, 2F, CF_2), -126.97 (s, 2F, CF_2).

^{13}C NMR ($CDCl_3$, 125.8 MHz): δ = 182.03 (s, 1C, C=O), 173.61 (t, 1C, J=26.6Hz, OH-C=), 139.28(s,1C, C_{Th}), 135.37 (s,1C, C_{Th}), 132.87 (s,1C, C_{Th}),128.96 (s,1C, C_{Th}), 95.14 (s, 1C, CH=). The signals of CF_2 and CF_3 groups were not fully acquired and were not resolved.

MS (EI, 70 eV): m/z (%) = 472(5.5) $[M]^+$, 153(100) $[M-C_6F_{13}]^+$, 111(35.5) $[ThCO]^+$, 69(37.5) $[CF_3]^+$.

HRMS (ESI+): m/z $[M]^+$ calcd for $C_{13}H_5F_{13}O_2S$: 471.9803; found: 471.9807.

Anal. Calcd for $C_{13}H_5F_{13}O_2S$: C,33.07; H, 1.07; F, 52.30. Found: C, 32.91; H, 1.28; F, 52.46.

4,4,5,5,6,6,7,7,8,8,9,9,10,10,10-Pentadecafluoro-1-(2-thienyl)decane-1,3-dione (3f)

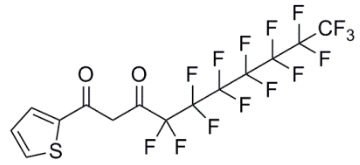

Yellow solid; yield: 20.1 g (77%); bp 122-125 °C (2 Torr); mp 53.5–54.5 °C.

IR (KBr): 1643; 1411; 1201; 1146; 1061; 1025; 809; 722; 673; 554; 527 cm^{-1}.

^{1}H NMR ($CDCl_3$, 300 MHz): δ = 14.95 (broad s, 1H, OH), 7.88 (d, 1H, J=3.7Hz, CH), 7.79(d, 1H, J=4.9Hz, CH), 7.23 (t, 1H, J=4.2Hz, CH), 6.50 (s, 1H, CH=C).

^{19}F NMR ($CDCl_3$, 282.5 MHz): δ = -81.64 (s, 3F, CF_3), -121.16 (t, 2F, J=11.3Hz, CF_2), -122.30 (s, 2F, CF_2), -122.76 (s, 2F, CF_2), -123.10 (s, 2F, CF_2), -123.51 (s, 2F, CF_2), -126.91 (s, 2F, CF_2).

^{13}C NMR ($CDCl_3$, 151 MHz): δ = 182.64 (s, 1C, C=O), 177.12 (t, 1C, J=26.4Hz, OH-C=), 139.85(s,1C, C_{Th}), 136.05 (s,1C, C_{Th}), 132.51 (s,1C, C_{Th}),129.60 (s,1C, C_{Th}), 118.79, 111.63,110.81 (m, 7C, C_7F_{15}), 95.75 (s, 1C, CH=). The signals of CF_2 and CF_3 groups were not fully acquired and were not resolved.

MS (EI, 70 eV): m/z (%) = 2.5(5.5) $[M]^+$, 153(100) $[M-C_7F_{15}]^+$, 111(36) $[ThCO]^+$, 69(32) $[CF_3]^+$.

HRMS (ESI+): m/z $[M]^+$ calcd for $C_{14}H_5F_{15}O_2S$: 521.9771; found: 521.9775.

Anal. Calcd for $C_{14}H_5F_{15}O_2S$: C,32.20; H, 0.97; F, 54.57. Found: C, 32.34; H, 0.91; F, 54.69.

4,4,5,5,6,6,7,7,8,8,9,9,10,10,11,11,11-Leptadecafluoro-1-(2-thienyl)undecane-1,3-dione (3g)

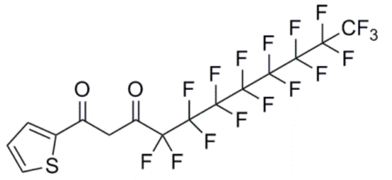

White solid; yield: 23.7 g (83%); bp 142-145 °C (2 Torr); mp 75–76 °C.

IR (KBr):1611;1409; 1202; 1151; 966; 794; 734; 672; 527 cm^{-1}.

^{1}H NMR ($CDCl_3$, 300 MHz): δ = 15.05(broad s, 1H, OH), 7.92 (d, 1H, J=3.7Hz, CH), 7.82(d, 1H, J=4.8Hz, CH), 7.23 (t, 1H, J=4.4Hz, CH), 6.51 (s, 1H, CH=C).

^{19}F NMR ($CDCl_3$, 282.5 MHz): δ = -81.64 (s, 3F, CF_3), -121.16 (t, 2F, J=11.3Hz, CF_2), -122.26 (s, 2F, CF_2), -122.64 (s, 4F, CF_2), -123.09 (s, 2F, CF_2), -123.50 (s, 2F, CF_2), -126.91 (s, 2F, CF_2).

^{13}C NMR ($CDCl_3$, 151 MHz): δ = 182.67 (s, 1C, C=O), 174.13 (m, 1C, OH-C=), 139.86(s,1C, C_{Th}), 136.04 (s,1C, C_{Th}), 133.50 (s,1C, C_{Th}),129.60 (s,1C, C_{Th}), 119.96, 114.91,111.54, 108.57 (m, 7C, C_7F_{15}), 95.75 (s, 1C, CH=). The signals of CF_2 and CF_3 groups were not fully acquired and were not resolved.

MS (EI, 70 eV): m/z (%) = 572(1.5) $[M]^+$, 153(100) $[M-C_8F_{17}]^+$, 111(30.5) $[ThCO]^+$, 69(30) $[CF_3]^+$.

HRMS (ESI+): m/z $[M]^+$ calcd for $C_{15}H_5F_{17}O_2S$: 571.9739; found: 571.97736.

Anal. Calcd for $C_{15}H_5F_{17}O_2S$: C,31.48; H, 0.88; F, 56.44. Found: C, 31.26; H, 0.81; F, 56.09.

1-(2-Thienyl)butane-1,3-dione (5)

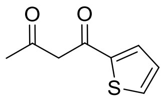

Yellow solid; yield: 3.6 g (71%); bp 105-106 °C (3 Torr); mp 35–36 °C (lit. 35-37)[3].

IR (KBr): 1721, 1614, 1520, 1411, 1325, 1277, 1235, 793, 721, 621 cm^{-1}.

^{1}H NMR ($CDCl_3$, 300 MHz, a mixture of keto- and enol forms): δ = 16.65 (broad s, 1H, OH), 7.69 (m, 1.31 H, CH), 7.61 (m, 0.92 H, CH), 7.17 (m, 1.13 H, CH), 6.03(s, 1 H, CH=C enol form), 4.03(s, 0.40 H, CH_2, keto form), 2.31(s, 0.64 H, CH_3 enol form), 2.14 (s, 3 H, CH_3 keto form).

^{13}C NMR ($CDCl_3$, 75.5 MHz, mixture of keto- and enol forms): δ = 201.41, 187.43, 186.14, 181.71, 143.68, 141.74, 135.12 (keto form), 133.75 (keto form), 132.31, 130.20, 128.53 (keto form), 128.23, 96.41, 55.44 (keto form), 30.42, 23.64 (keto form).

MS (EI, 70 eV): m/z (%) = 168 (100) $[M]^+$, 135(32) $[M\text{-}SH]^+$, 111(68) $[ThCO]^+$, 43(26) $[CH_3\text{-}CO]^+$.

HRMS (ESI+): m/z $[M + Na]^+$ calcd for $C_8H_8O_2S$: 191.0143; found: 191.0146.

References

1. Moore, R.A.; Levine, R. J*. Org. Chem.* **1964**, *29*, 1439.
2. Barkley, L. B.; Levine, R. *JACS* **1951**, *73*, 4625.
3. Sneed, J. K.; Levine, R. *JACS* **1950**, *72*, 5219.
