# Supplementary material for: A convenient and practical synthesis of β-diketones bearing linear perfluorinated alkyl groups and a 2-thienyl moiety
Source: Beilstein J Org Chem. 2018 Dec 27;14:3106–11. doi: 10.3762/bjoc.14.290 (PMC6317410; doi:10.3762/bjoc.14.290)
Supplement: File 2 — Copies of 19F and 13C NMR spectra and LR mass spectra of compounds 3a–g and 5. [file Beilstein_J_Org_Chem-14-3106-s002.pdf]

BEILSTEIN JOURNAL OF ORGANIC CHEMISTRY

Supporting Information

for

A convenient and practical synthesis of β-diketones bearing linear perfluorinated alkyl groups and a 2-thienyl moiety

Ilya V. Taydakov, Yuliya M. Kreshchenova and Ekaterina P. Dolotova

Beilstein J. Org. Chem. **2018,** *14,* 3106–3111. doi:10.3762/bjoc.14.290

Copies of ^{19}F and ^{13}C NMR spectra and LR mass spectra of compounds 3a–g and 5

License and Terms: This is a supporting information file under the terms of the Creative Commons Attribution License (http://creativecommons.org/licenses/by/4.0). Please note that the reuse, redistribution and reproduction in particular requires that the authors and source are credited.
The license is subject to the *Beilstein Journal of Organic Chemistry* terms and conditions: (https://www.beilstein-journals.org/bjoc)

Table of Contents:

^{13}C and ^{19}F NMR spectra of compounds **3a–g** S2 – S15
^{1}H and ^{13}C NMR spectra of compound **5** S16 – S17
EI (70eV) ionization mass-spectra of compounds **3a–g** and **5** S18 – S25

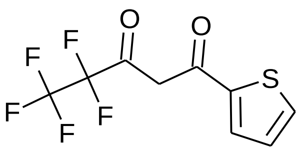

3a

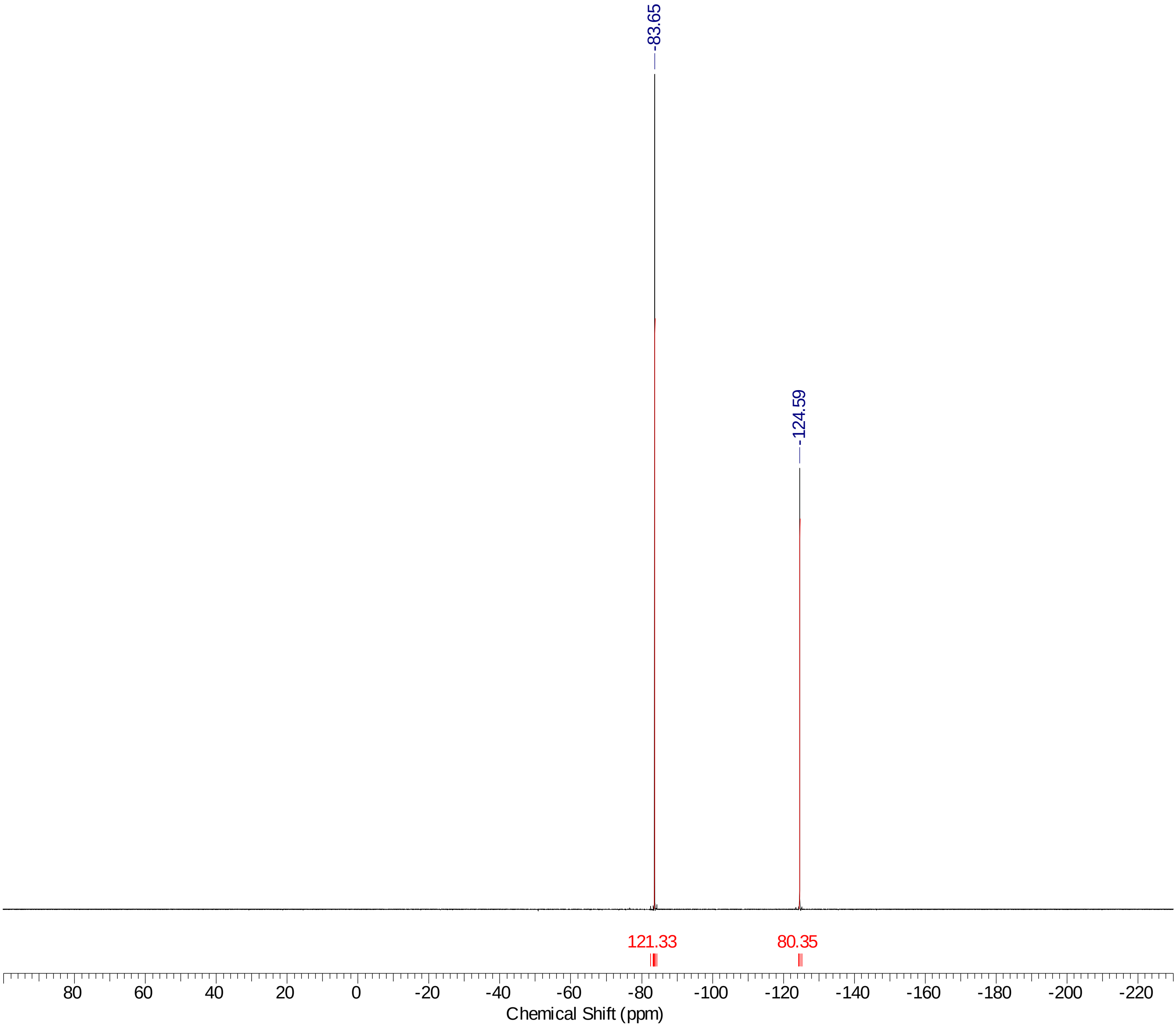
-83.65
-124.59
121.33
80.35
80
60
40
20
0
-20
-40
-60
-80
-100
-120
-140
-160
-180
-200
-220
Chemical Shift (ppm)

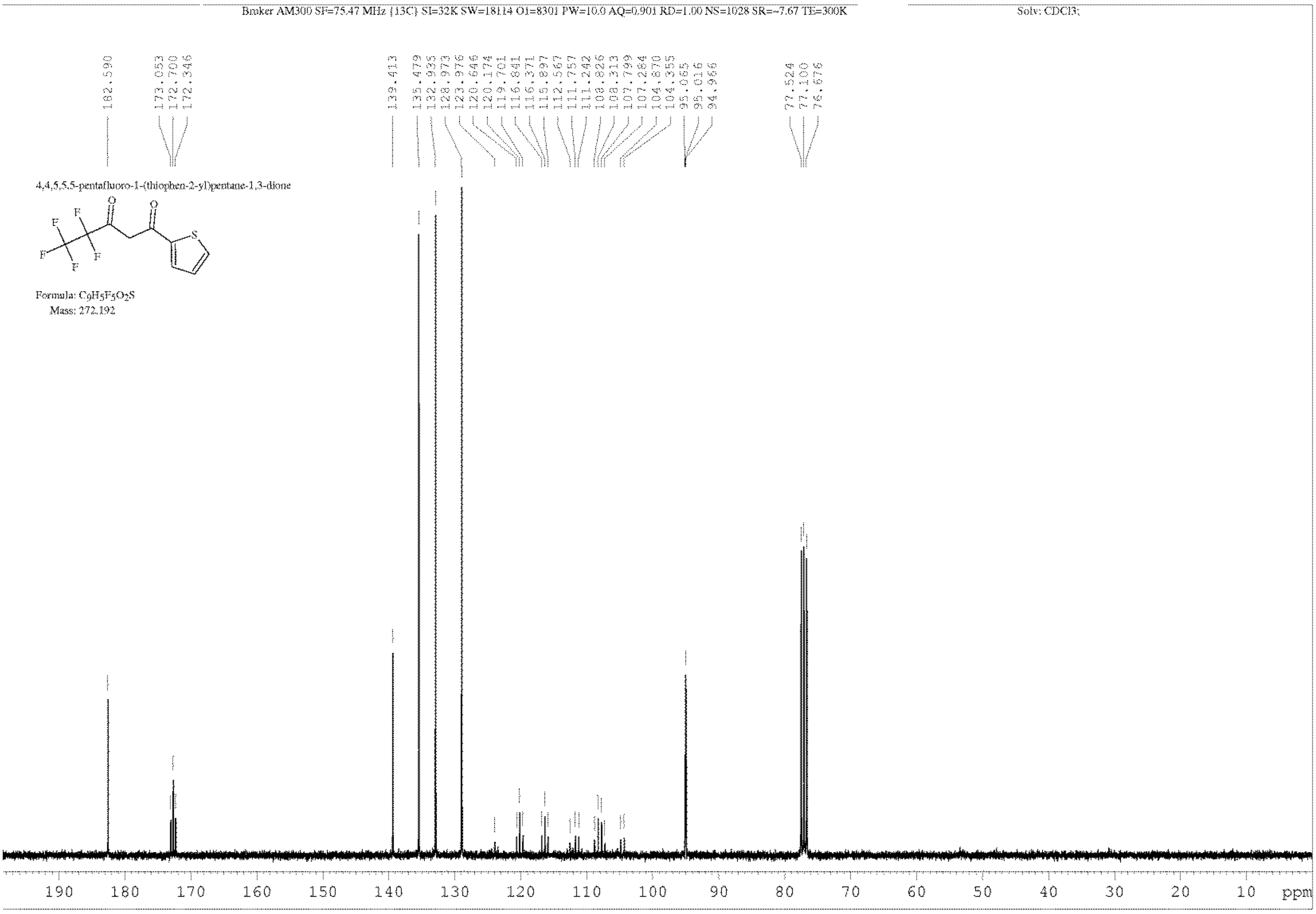
Bruker AM300 SF=75.47 MHz {13C} SI=32K SW=18114 O1=8301 PW=10.0 AQ=0.901 RD=1.00 NS=1028 SR=-7.67 TE=300K
Solv: CDCl3;
182.590
173.053
172.700
172.346
139.413
135.479
132.935
128.973
123.976
120.646
120.174
119.701
116.841
116.371
115.897
112.567
111.757
111.242
108.826
108.313
107.799
107.284
104.870
104.355
95.065
95.016
94.966
77.524
77.100
76.676
4,4,5,5,5-pentafluoro-1-(thiophen-2-yl)pentane-1,3-dione
Formula: C9H5F5O2S
Mass: 272.192
190
180
170
160
150
140
130
120
110
100
90
80
70
60
50
40
30
20
10
ppm

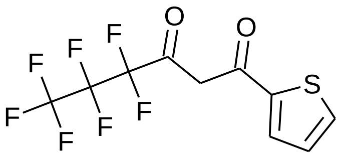

O
O
F
F
F
S
F
F
F
F

3b

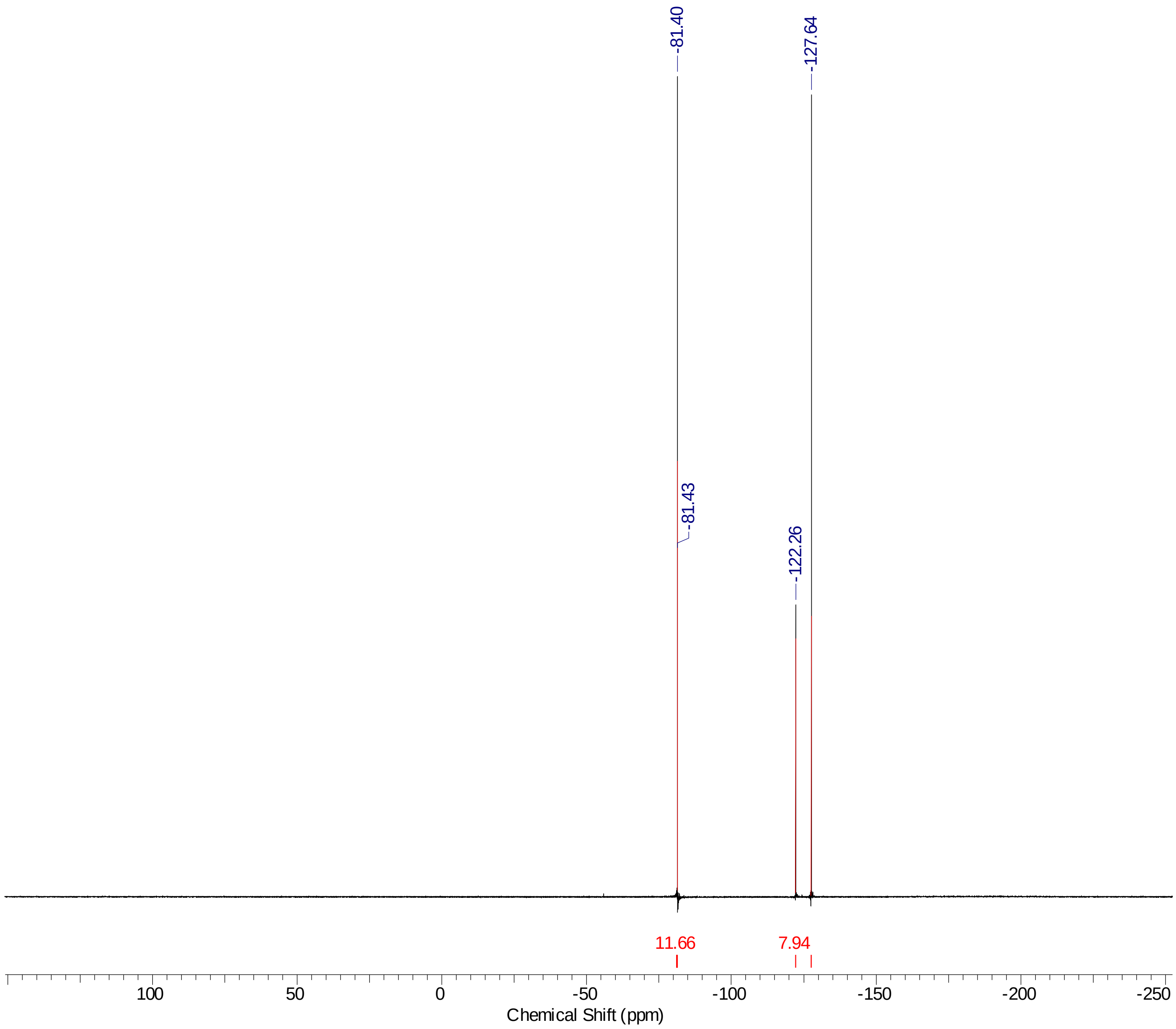

-81.40
-127.64
-81.43
-122.26
11.66
7.94
100
50
0
-50
-100
-150
-200
-250
Chemical Shift (ppm)

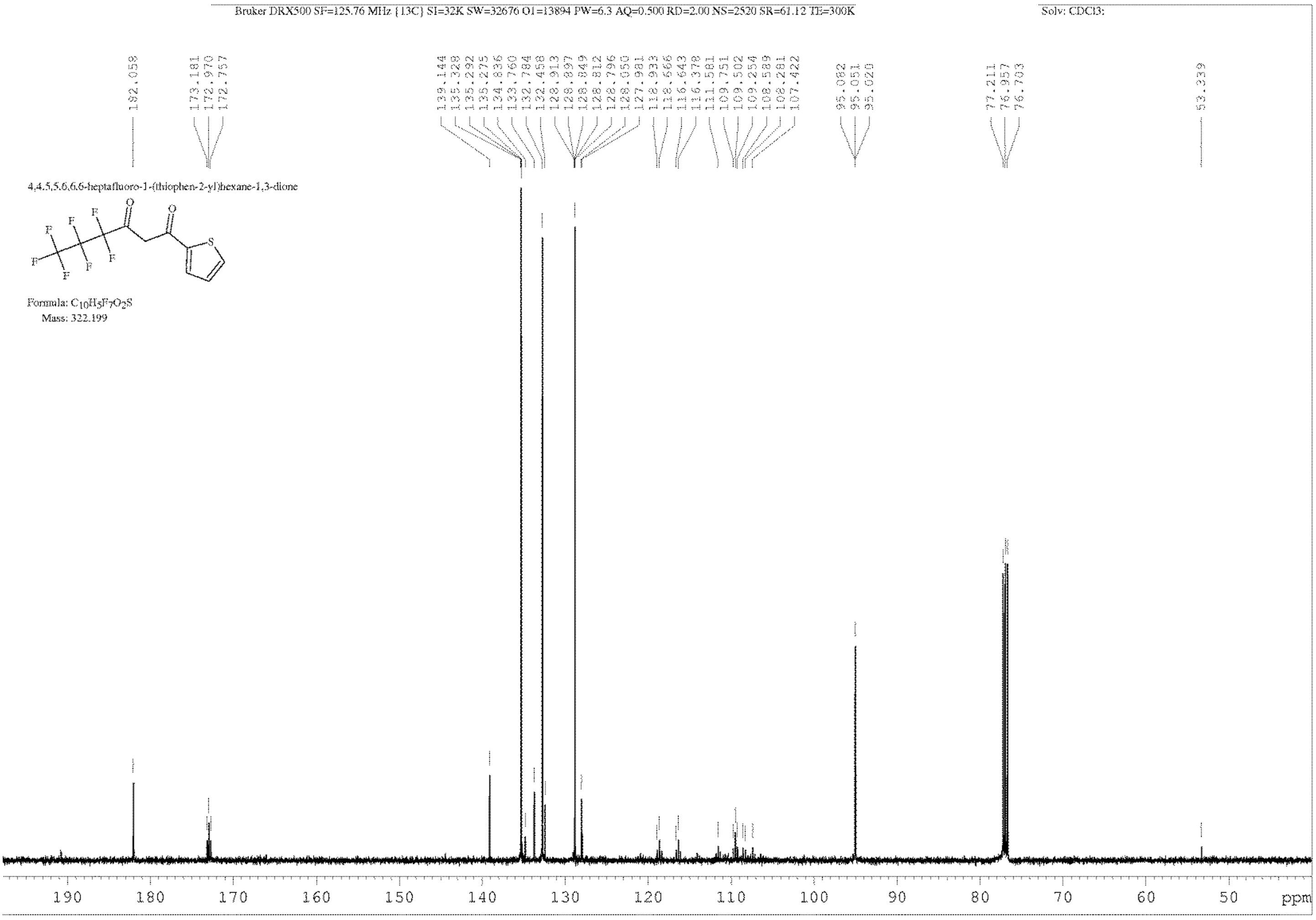
Bruker DRX500 SF=125.76 MHz {13C} SI=32K SW=32676 O1=13894 PW=6.3 AQ=0.500 RD=2.00 NS=2520 SR=61.12 TE=300K
Solv: CDCl3:
182.058
173.181
172.970
172.757
139.144
135.328
135.292
135.275
134.836
133.760
132.784
132.458
128.913
128.897
128.849
128.812
128.796
128.050
127.981
118.933
118.666
116.643
116.378
111.581
109.751
109.502
109.254
108.589
108.281
107.422
95.082
95.051
95.020
77.211
76.957
76.703
53.339
4,4,5,5,6,6,6-heptafluoro-1-(thiophen-2-yl)hexane-1,3-dione
Formula: C10H5F7O2S
Mass: 322.199
190
180
170
160
150
140
130
120
110
100
90
80
70
60
50
ppm

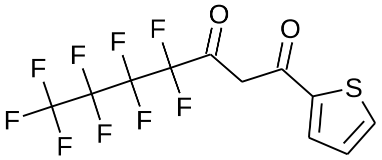

3c

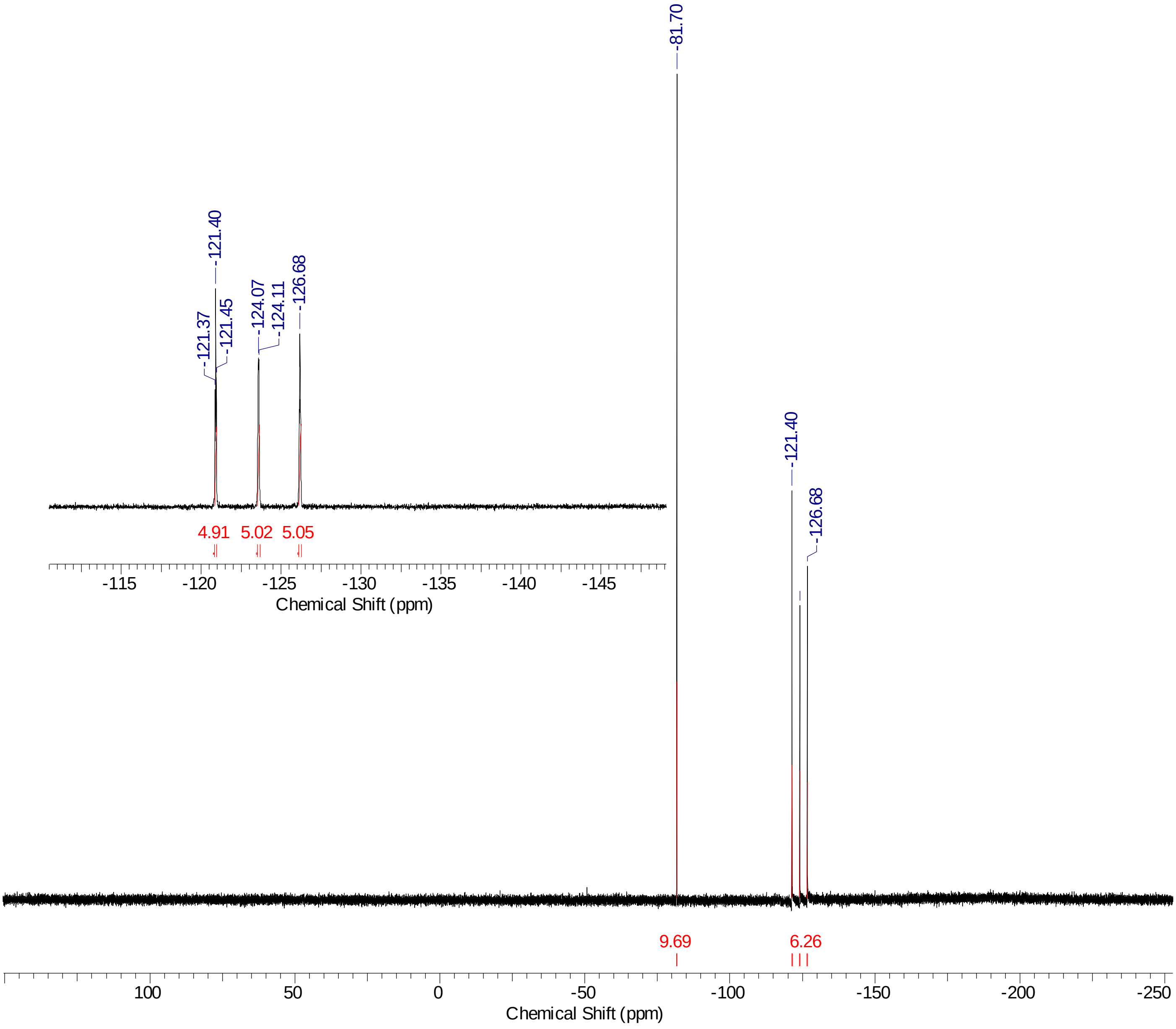
-81.70
-121.40
-126.68
-121.37
-121.40
-121.45
-124.07
-124.11
-126.68
4.91
5.02
5.05
-115
-120
-125
-130
-135
-140
-145
Chemical Shift (ppm)
9.69
6.26
100
50
0
-50
-100
-150
-200
-250
Chemical Shift (ppm)

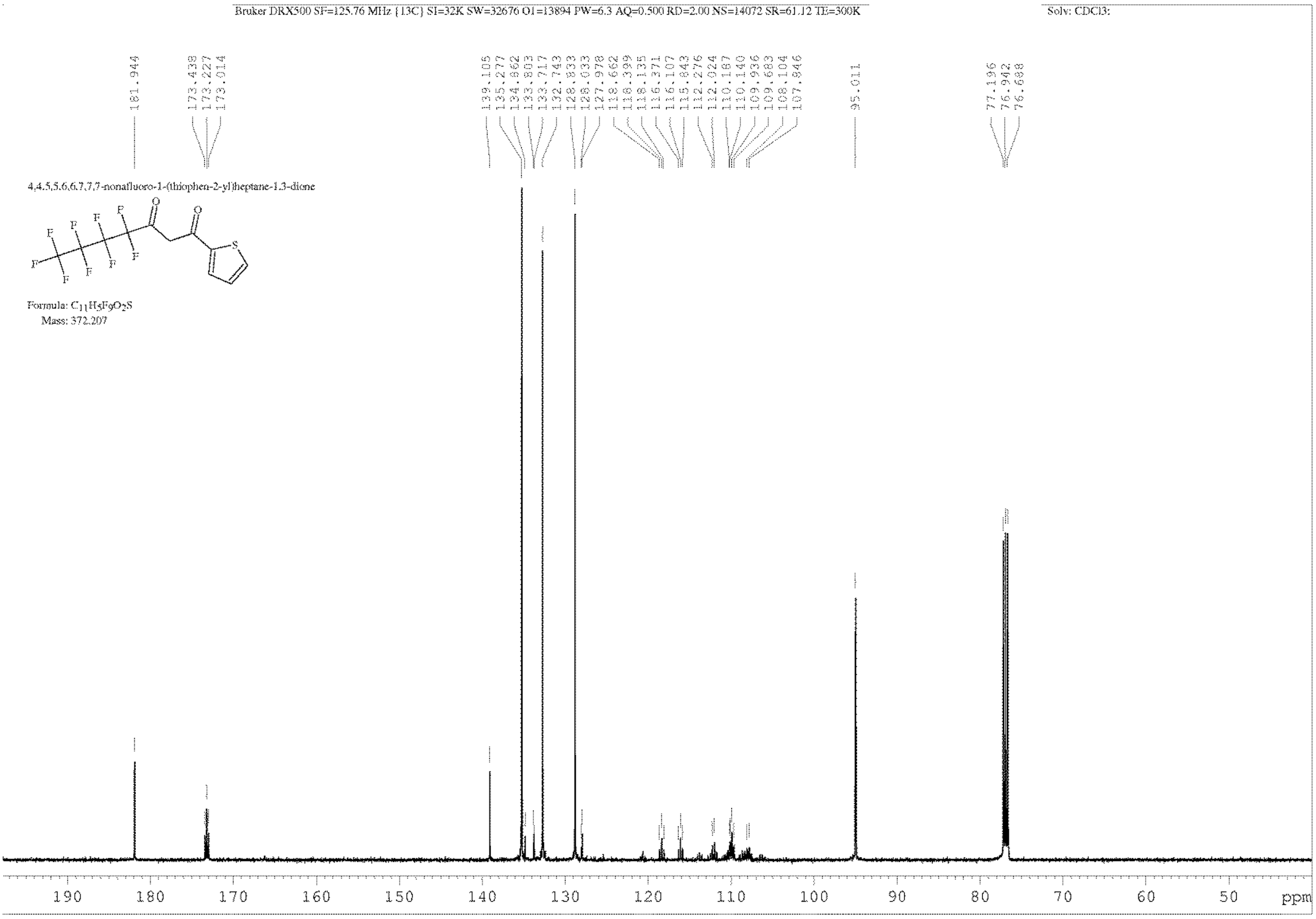
Bruker DRX500 SF=125.76 MHz {13C} SI=32K SW=32676 O1=13894 PW=6.3 AQ=0.500 RD=2.00 NS=14072 SR=61.12 TE=300K
Solv: CDCl3:
181.944
173.438
173.227
173.014
139.105
135.277
134.862
133.803
133.717
132.743
128.833
128.033
127.978
118.662
118.399
118.135
116.371
116.107
115.843
112.276
112.024
110.187
110.140
109.936
109.683
108.104
107.846
95.011
77.196
76.942
76.688
4,4,5,5,6,6,7,7,7-nonafluoro-1-(thiophen-2-yl)heptane-1,3-dione
Formula: C11H5F9O2S
Mass: 372.207
190
180
170
160
150
140
130
120
110
100
90
80
70
60
50
ppm

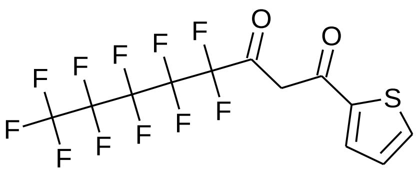
O
O
S
F
F
F
F
F
F
F
F
F
F
F
F
F

3d

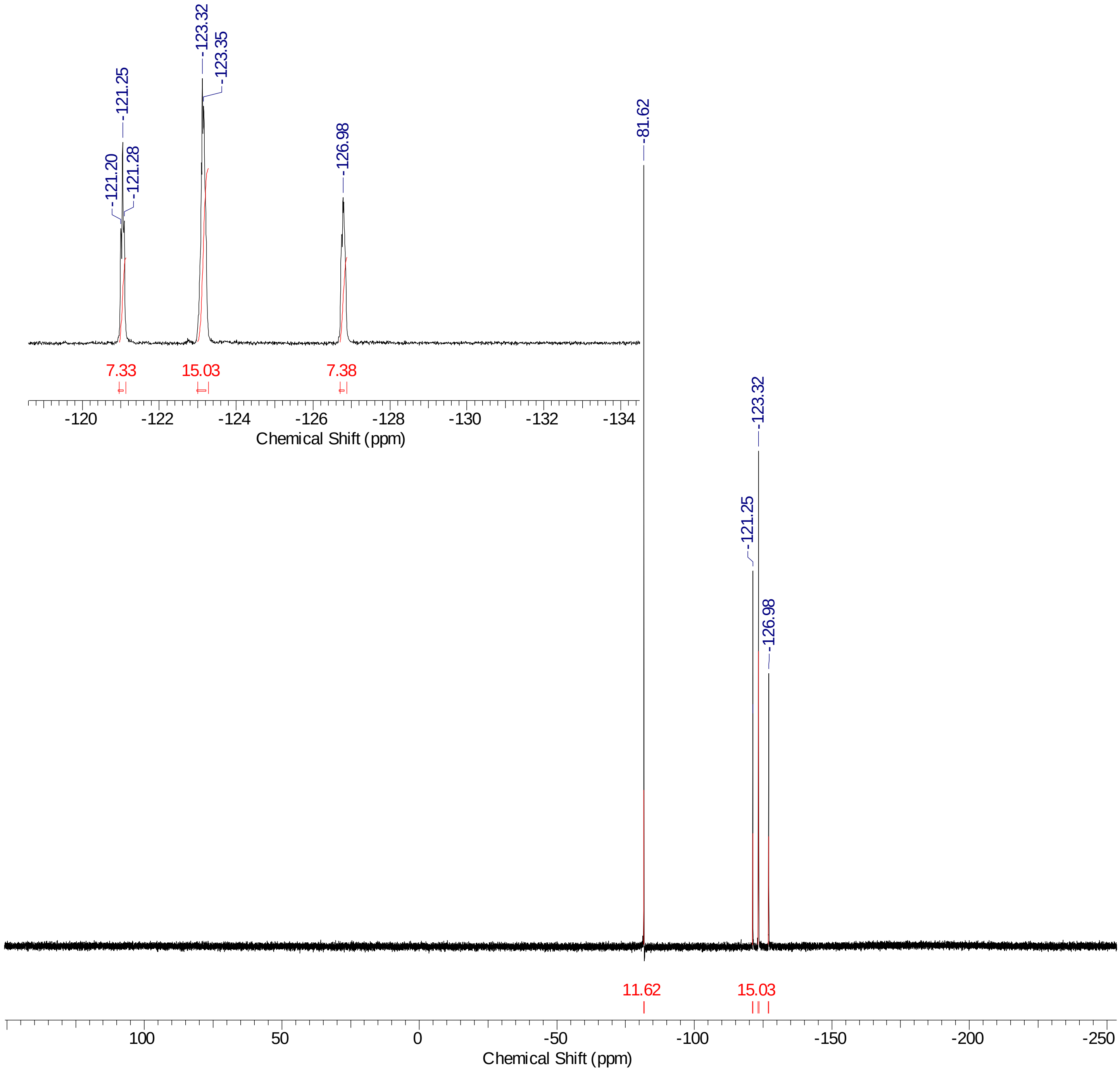
-121.20
-121.25
-121.28
-123.32
-123.35
-126.98
-81.62
7.33
15.03
7.38
-120
-122
-124
-126
-128
-130
-132
-134
Chemical Shift (ppm)
-123.32
-121.25
-126.98
11.62
15.03
100
50
0
-50
-100
-150
-200
-250
Chemical Shift (ppm)

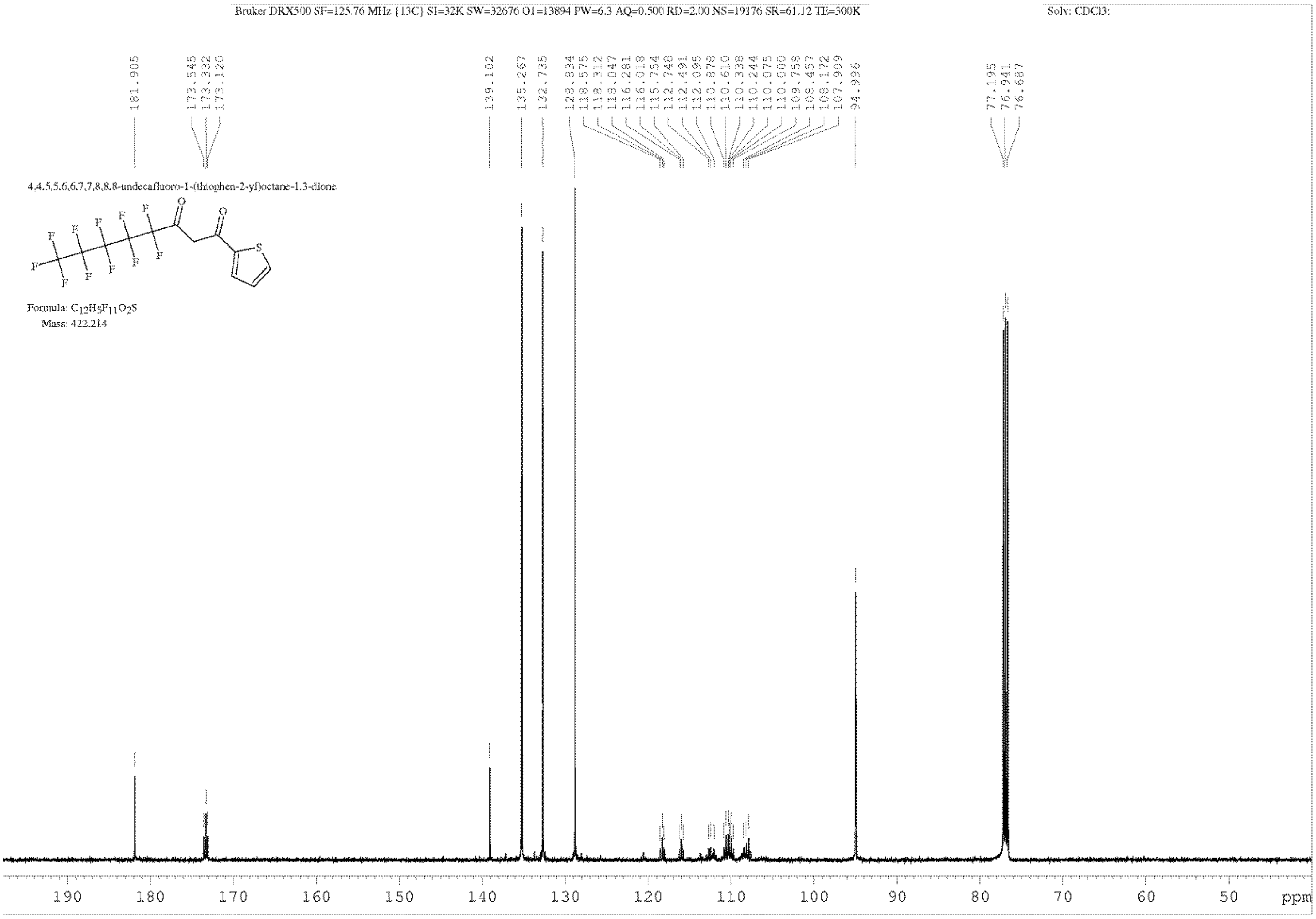

Bruker DRX500 SF=125.76 MHz {13C} SI=32K SW=32676 O1=13894 PW=6.3 AQ=0.500 RD=2.00 NS=19176 SR=61.12 TE=300K
Solv: CDCl3:
181.905
173.545
173.332
173.120
139.102
135.267
132.735
128.834
118.575
118.312
118.047
116.281
116.018
115.754
112.748
112.491
112.095
110.878
110.610
110.338
110.244
110.075
110.000
109.758
108.457
108.172
107.909
94.996
77.195
76.941
76.687
4,4,5,5,6,6,7,7,8,8,8-undecafluoro-1-(thiophen-2-yl)octane-1,3-dione
Formula: C12H5F11O2S
Mass: 422.214
190
180
170
160
150
140
130
120
110
100
90
80
70
60
50
ppm

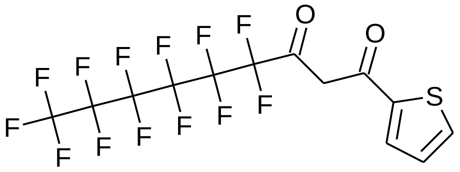
F
O
S

3e

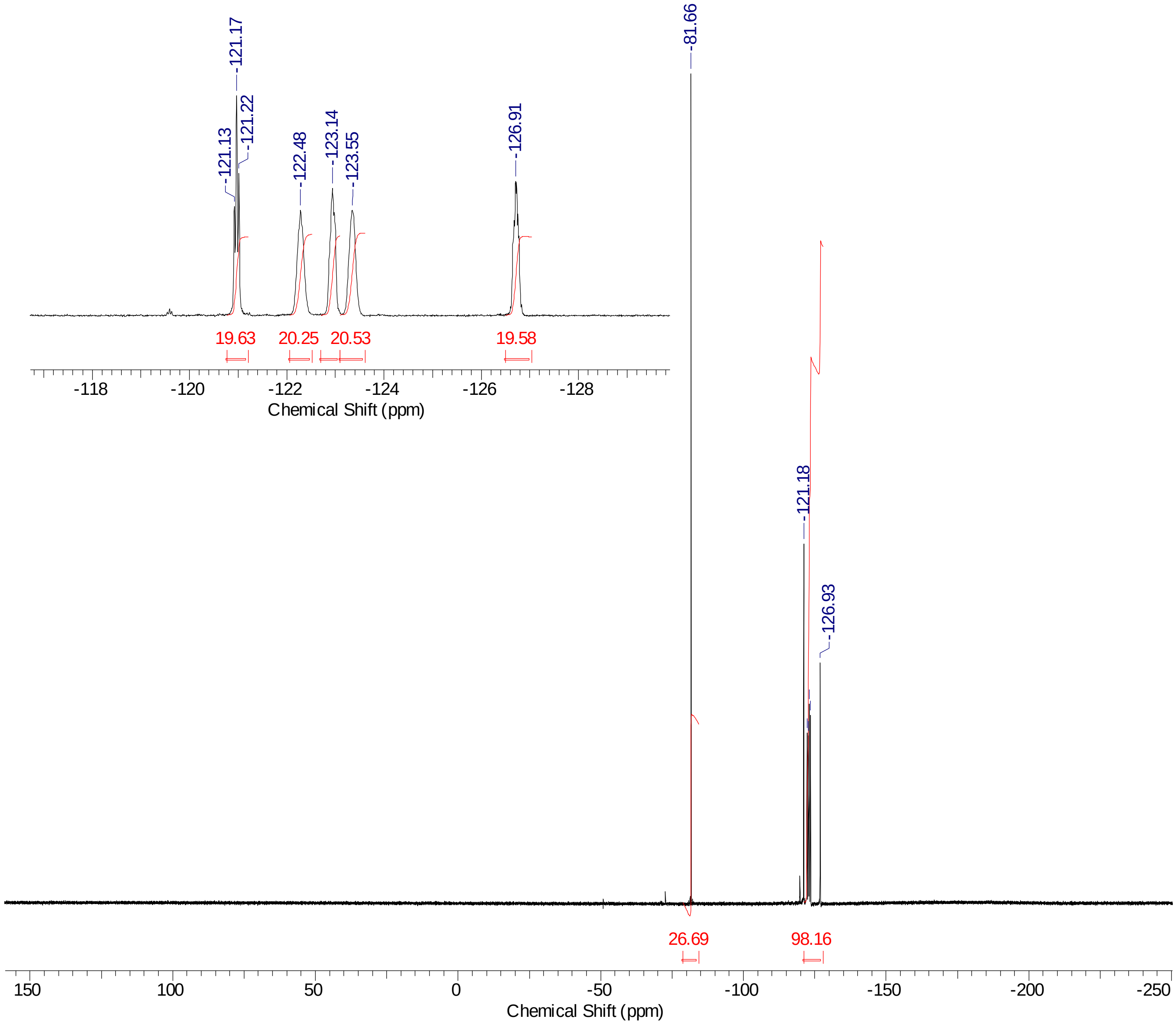
-121.17
-121.13
-121.22
-122.48
-123.14
-123.55
-126.91
19.63
20.25
20.53
19.58
-118
-120
-122
-124
-126
-128
Chemical Shift (ppm)
-81.66
-121.18
-126.93
26.69
98.16
150
100
50
0
-50
-100
-150
-200
-250
Chemical Shift (ppm)

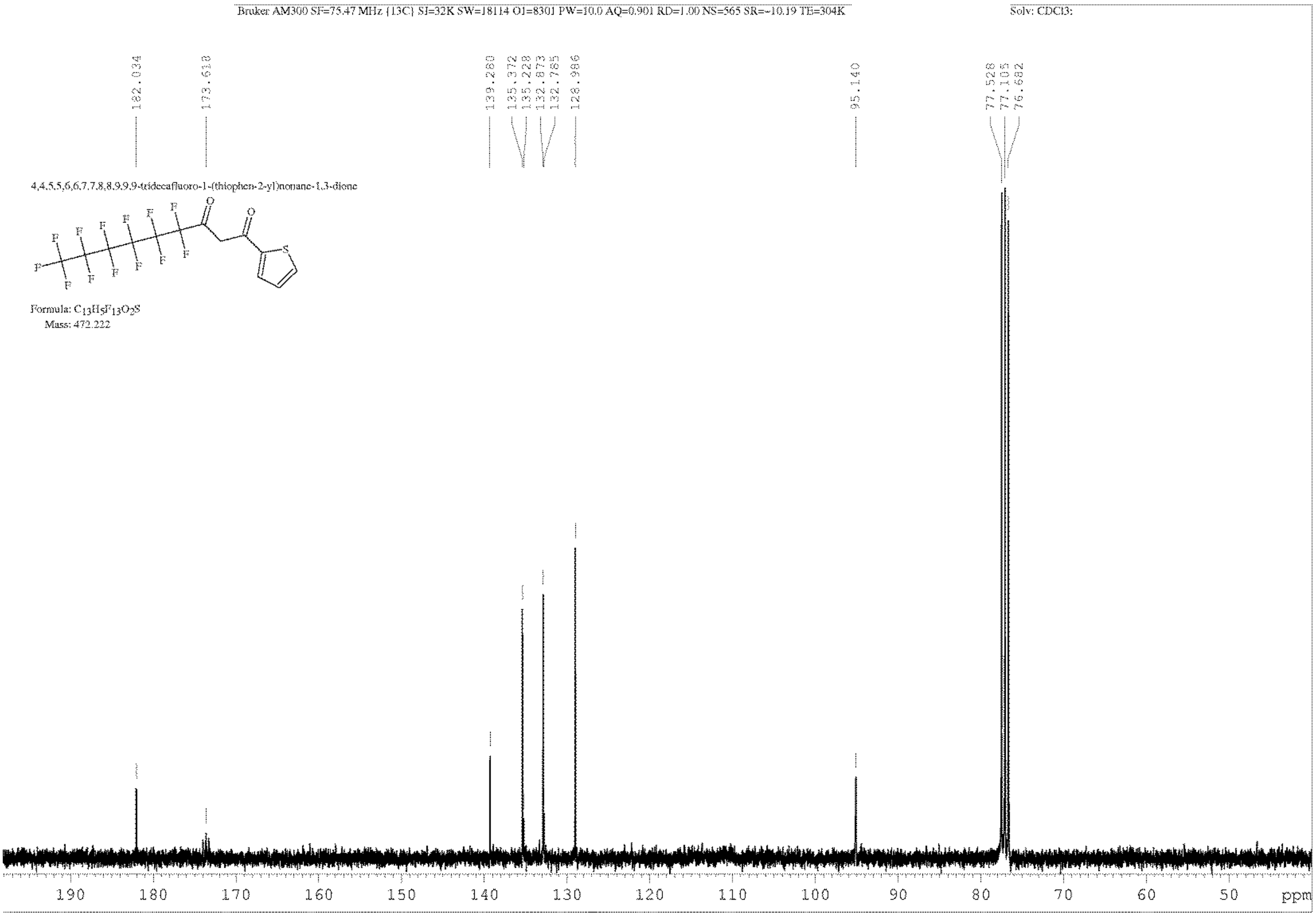
Bruker AM300 SF=75.47 MHz {13C} SI=32K SW=18114 O1=8301 PW=10.0 AQ=0.901 RD=1.00 NS=565 SR=-10.19 TE=304K
Solv: CDCl3:
182.034
173.618
139.280
135.372
135.228
132.873
132.785
128.986
95.140
77.528
77.105
76.682
4,4,5,5,6,6,7,7,8,8,9,9,9-tridecafluoro-1-(thiophen-2-yl)nonane-1,3-dione
Formula: C13H5F13O2S
Mass: 472.222
190 180 170 160 150 140 130 120 110 100 90 80 70 60 50 ppm

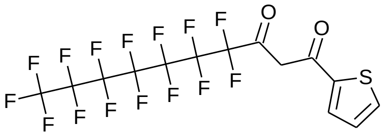
F
O
S

3f

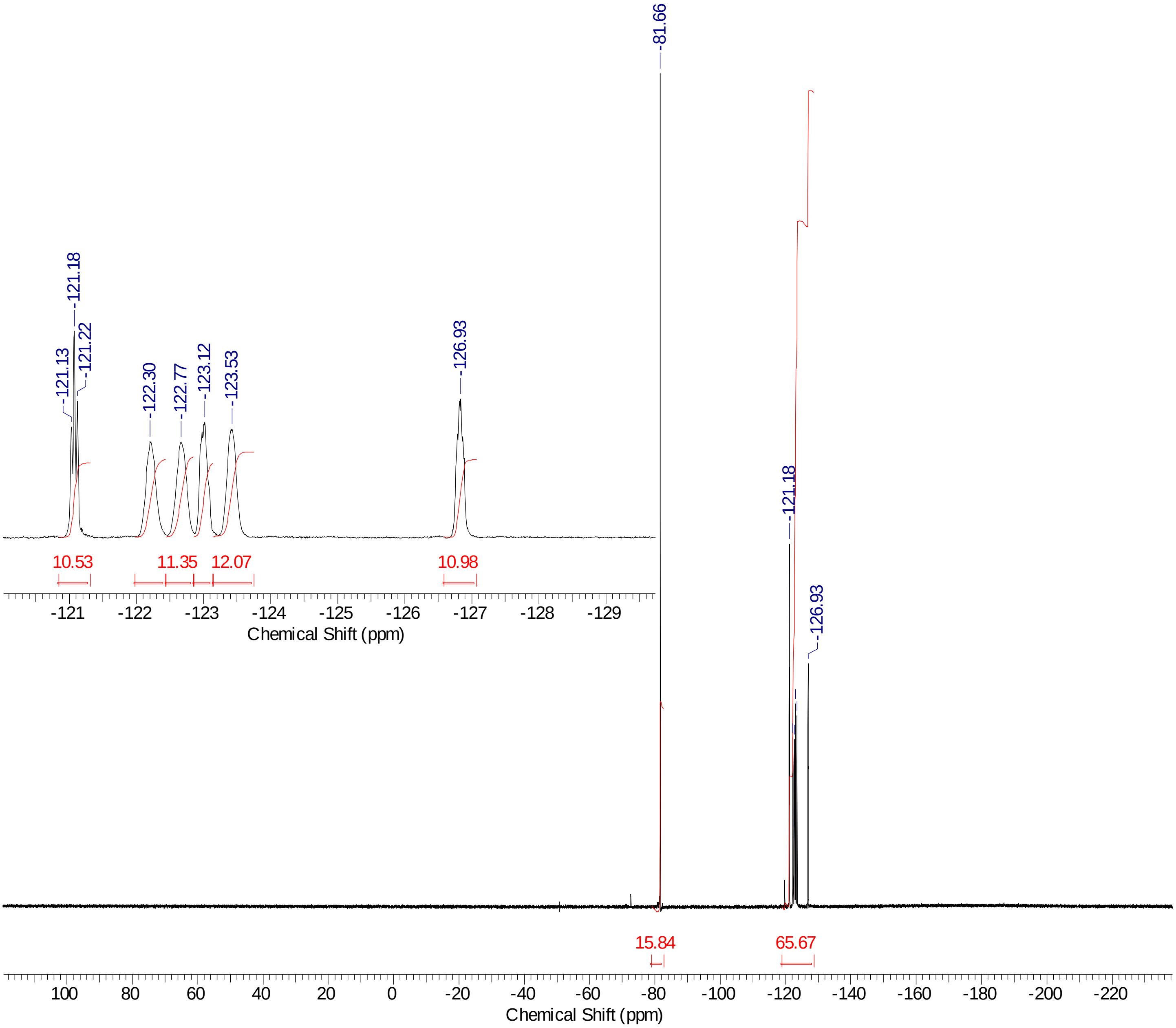
-81.66
-121.13
-121.18
-121.22
-122.30
-122.77
-123.12
-123.53
-126.93
10.53
11.35
12.07
10.98
-121
-122
-123
-124
-125
-126
-127
-128
-129
Chemical Shift (ppm)
-121.18
-126.93
15.84
65.67
100
80
60
40
20
0
-20
-40
-60
-80
-100
-120
-140
-160
-180
-200
-220
Chemical Shift (ppm)

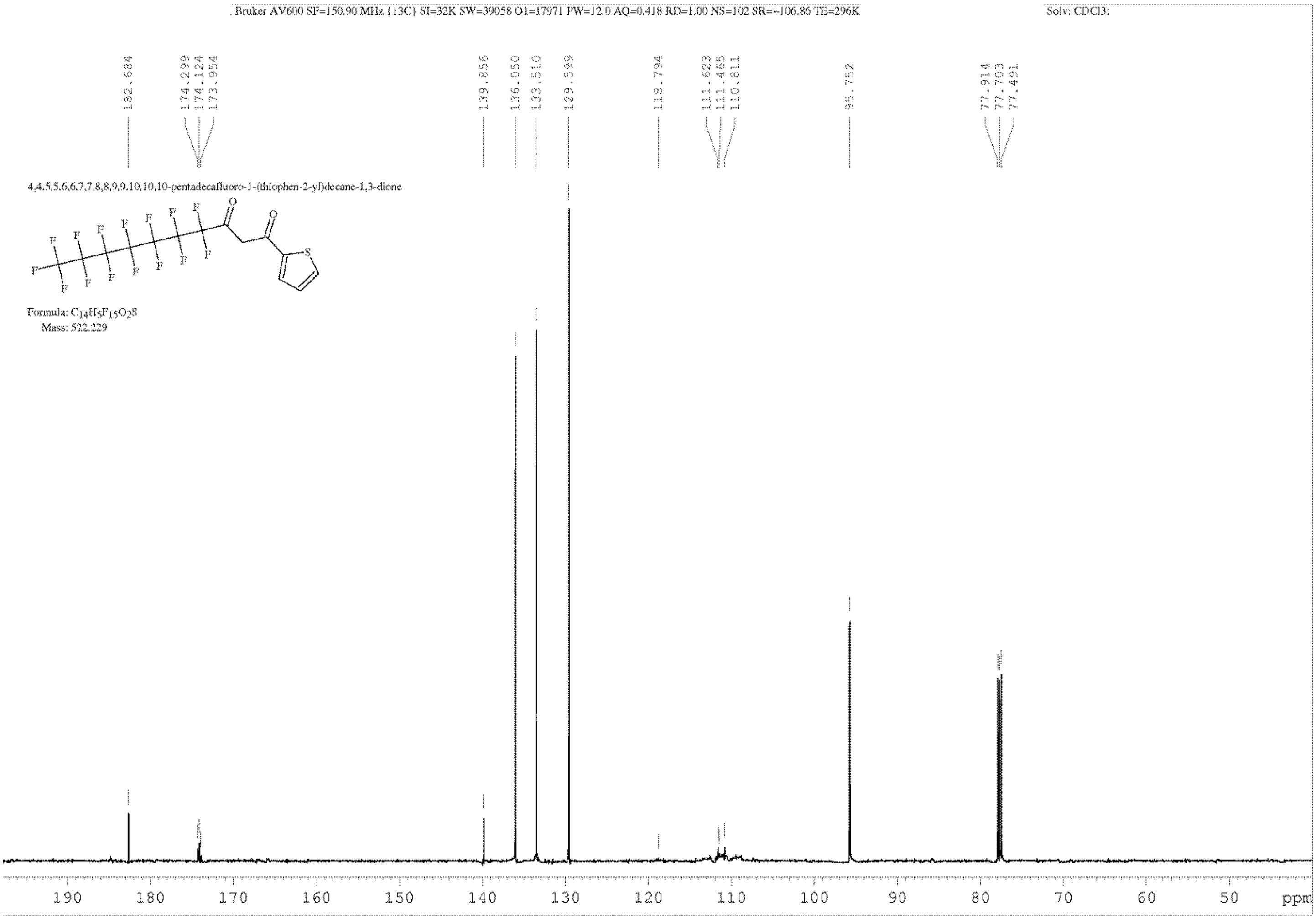
Bruker AV600 SF=150.90 MHz {13C} SI=32K SW=39058 O1=17971 PW=12.0 AQ=0.418 RD=1.00 NS=102 SR=-106.86 TE=296K
Solv: CDCl3:
182.684
174.299
174.124
173.954
139.856
136.050
133.510
129.599
118.794
111.623
111.465
110.811
95.752
77.914
77.703
77.491
4,4,5,5,6,6,7,7,8,8,9,9,10,10,10-pentadecafluoro-1-(thiophen-2-yl)decane-1,3-dione
Formula: C14H5F15O2S
Mass: 522.229
190
180
170
160
150
140
130
120
110
100
90
80
70
60
50
ppm

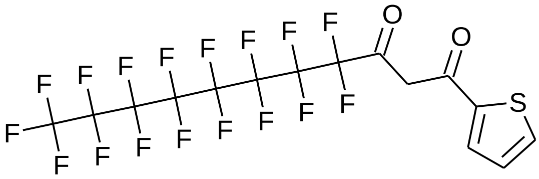

3g

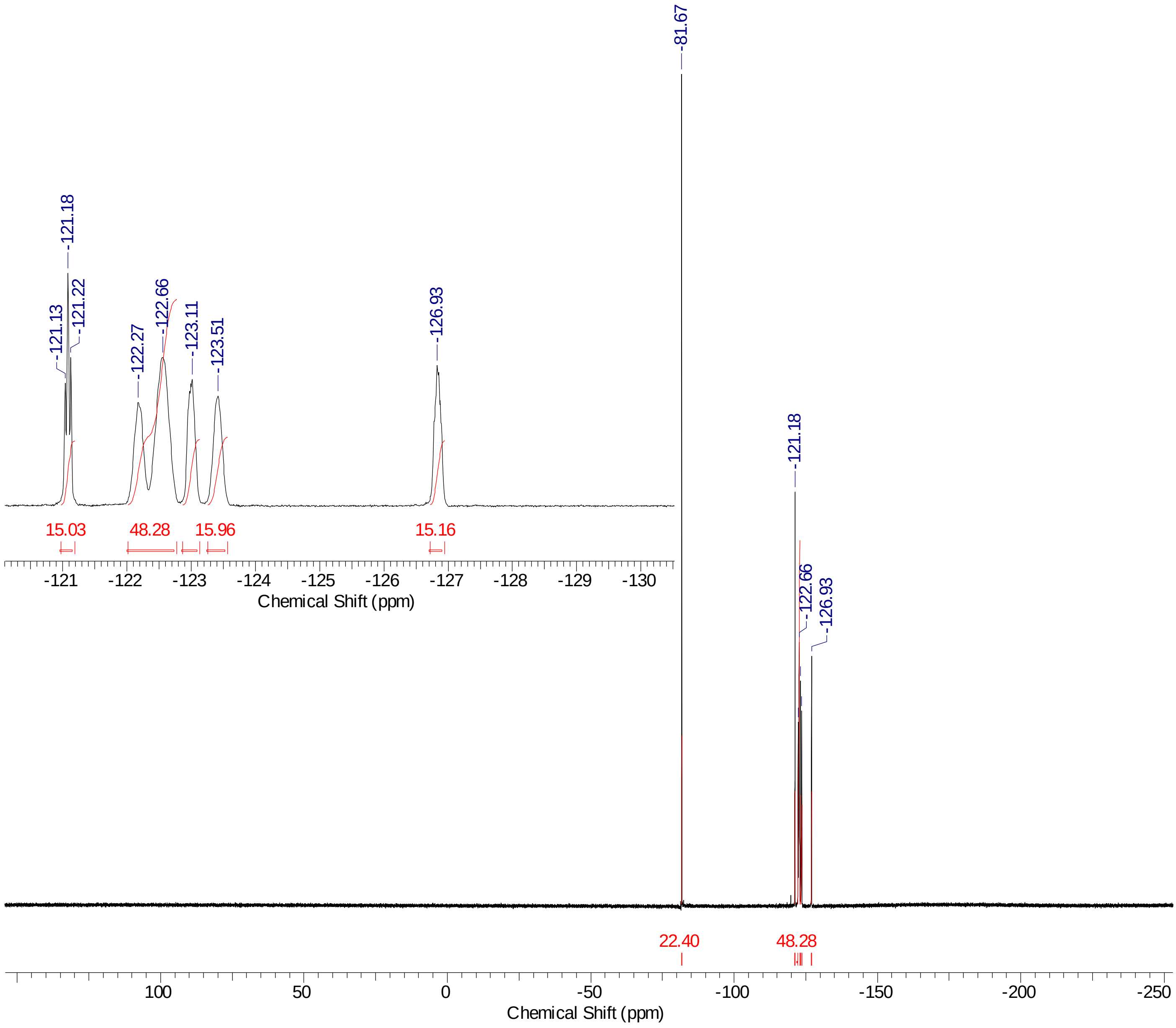

-81.67
-121.18
-121.13
-121.22
-122.27
-122.66
-123.11
-123.51
-126.93
15.03
48.28
15.96
15.16
-121 -122 -123 -124 -125 -126 -127 -128 -129 -130
Chemical Shift (ppm)
22.40
48.28
100 50 0 -50 -100 -150 -200 -250
Chemical Shift (ppm)

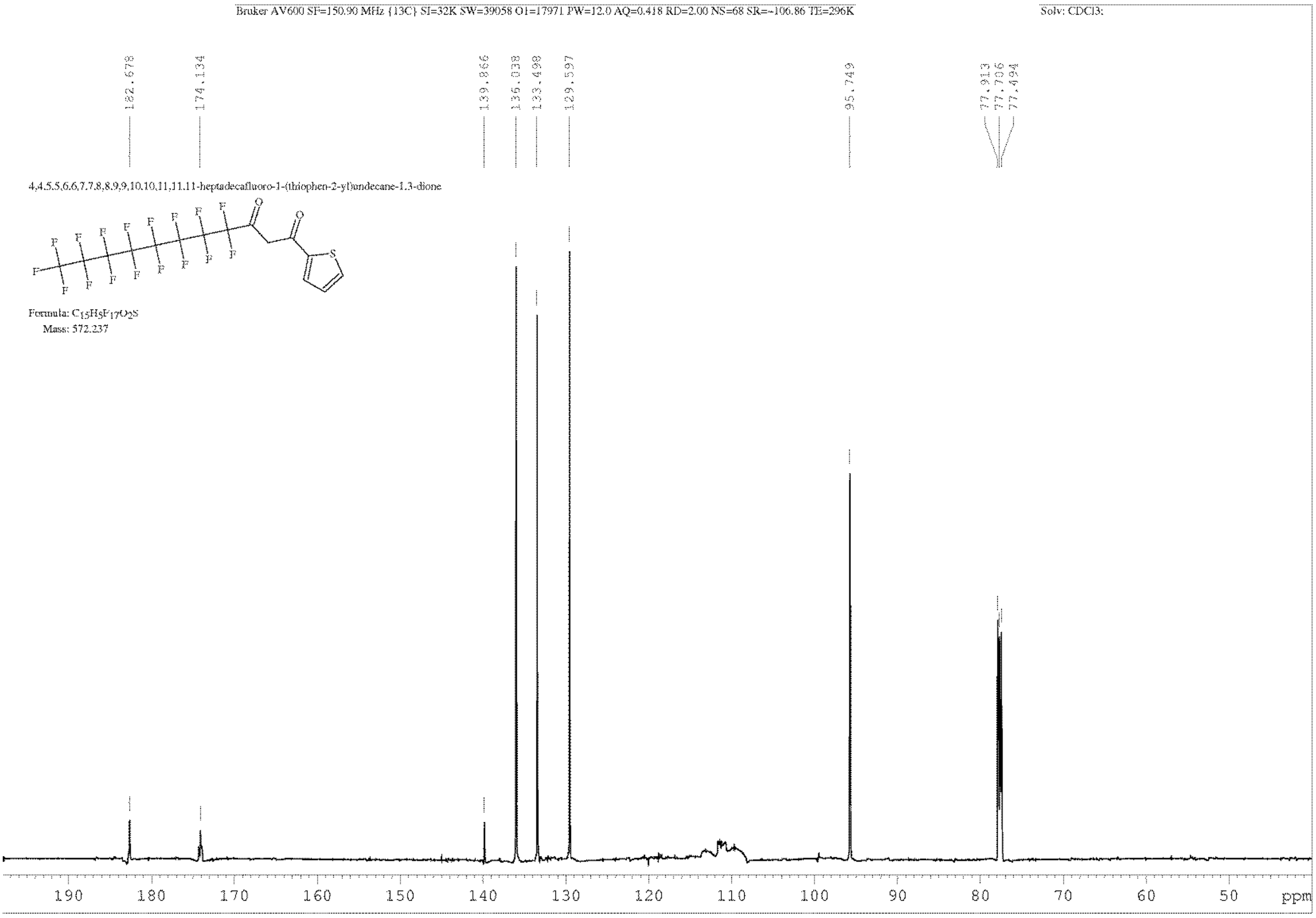
Bruker AV600 SF=150.90 MHz {13C} SI=32K SW=39058 O1=17971 PW=12.0 AQ=0.418 RD=2.00 NS=68 SR=-106.86 TE=296K
Solv: CDCl3;
182.678
174.134
139.866
136.038
133.498
129.597
95.749
77.913
77.706
77.494
4,4,5,5,6,6,7,7,8,8,9,9,10,10,11,11,11-heptadecafluoro-1-(thiophen-2-yl)undecane-1,3-dione
Formula: C15H5F17O2S
Mass: 572.237
190 180 170 160 150 140 130 120 110 100 90 80 70 60 50 ppm

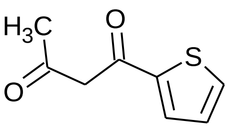

H3C
O
O
S

5

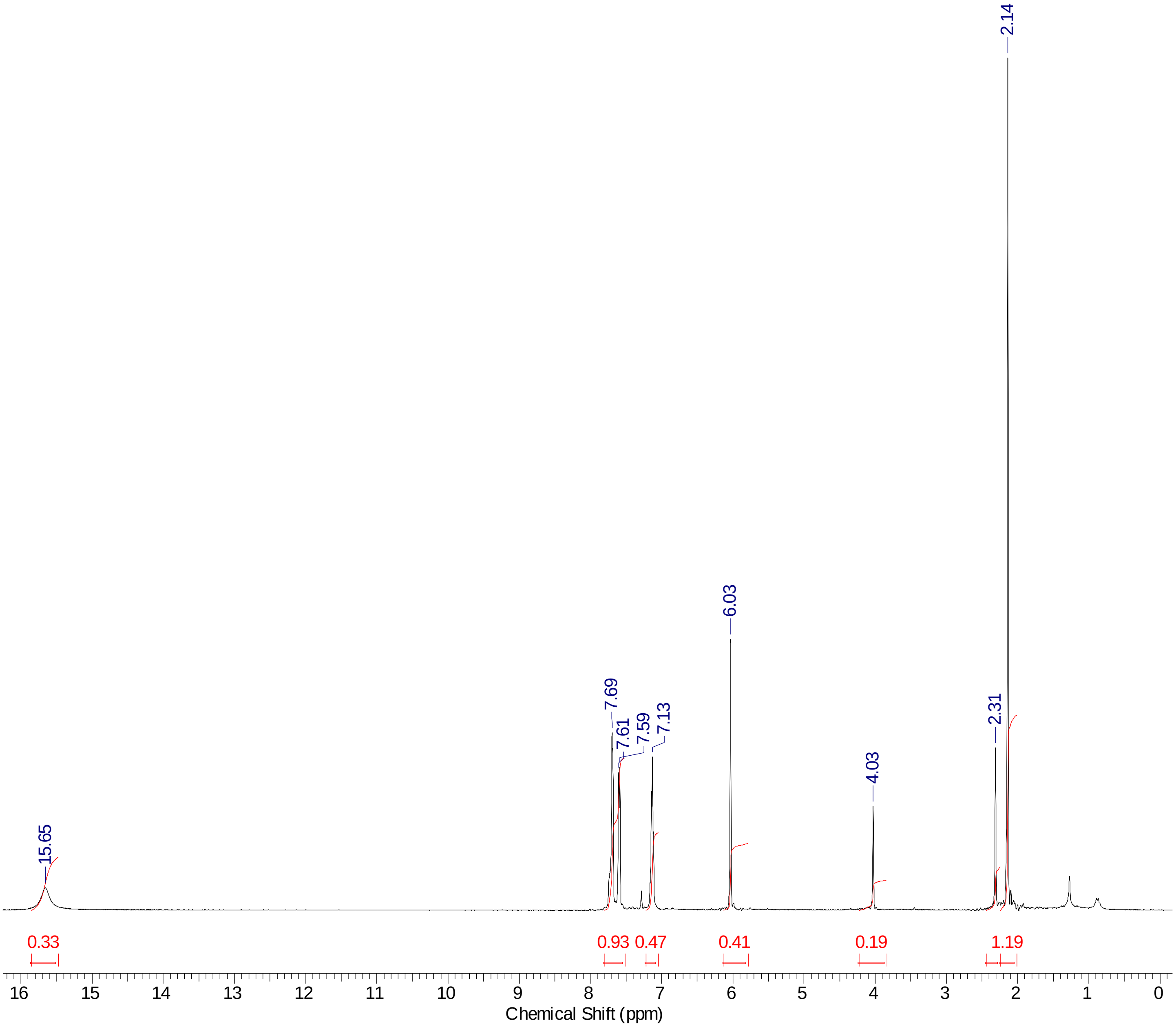

15.65
7.69
7.61
7.59
7.13
6.03
4.03
2.31
2.14
0.33
0.93
0.47
0.41
0.19
1.19
16 15 14 13 12 11 10 9 8 7 6 5 4 3 2 1 0
Chemical Shift (ppm)

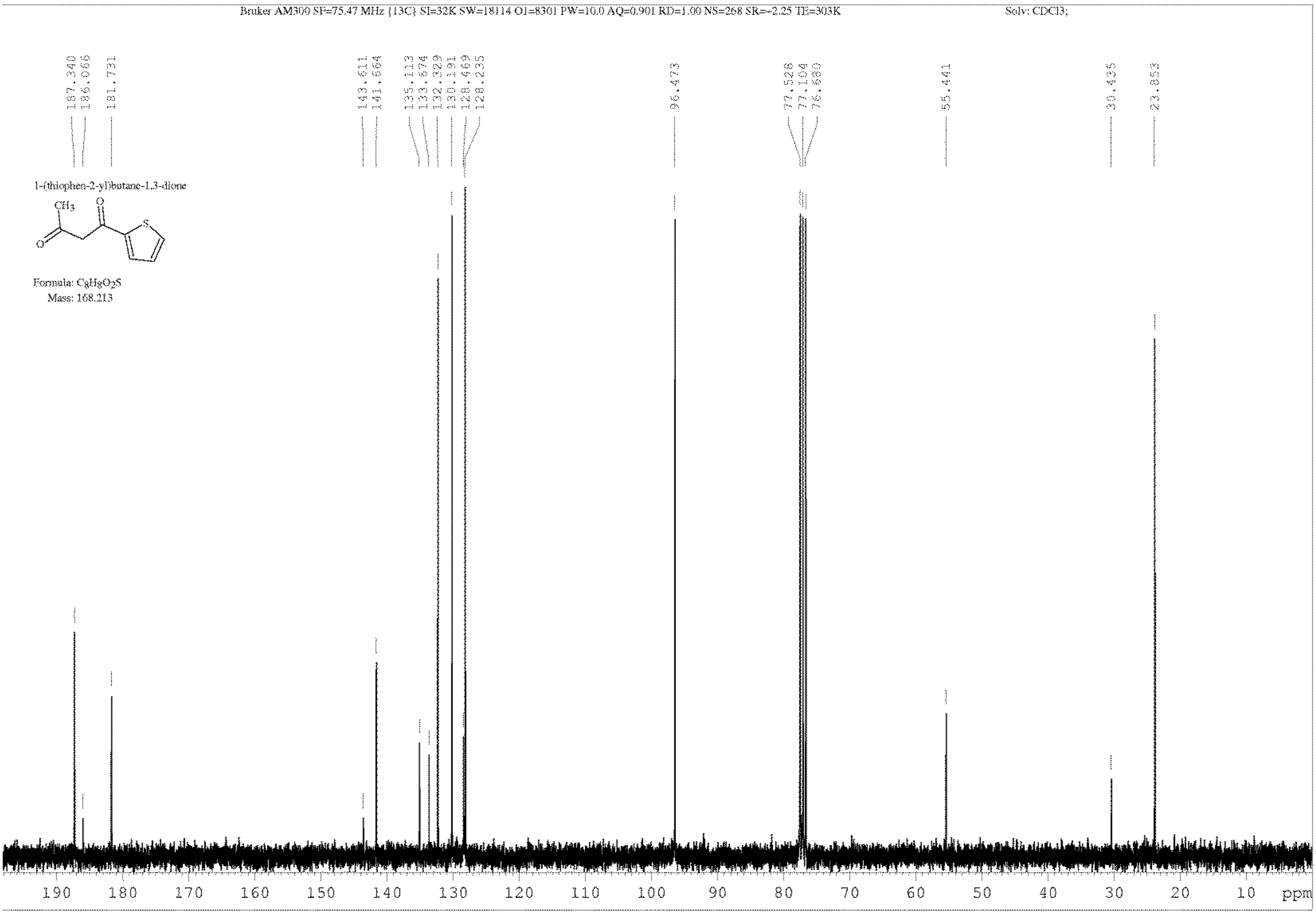
Bruker AM300 SF=75.47 MHz {13C} SI=32K SW=18114 O1=8301 PW=10.0 AQ=0.901 RD=1.00 NS=268 SR=-2.25 TE=303K
Solv: CDCl3;
187.340
186.066
181.731
143.611
141.664
135.113
133.674
132.329
130.191
128.469
128.235
96.473
77.528
77.104
76.680
55.441
30.435
23.853
1-(thiophen-2-yl)butane-1,3-dione
Formula: C8H8O2S
Mass: 168.213
190
180
170
160
150
140
130
120
110
100
90
80
70
60
50
40
30
20
10
ppm

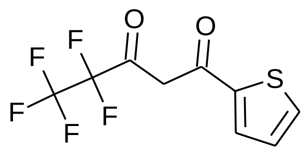

O
O
F
F
F
F
F
S

3a

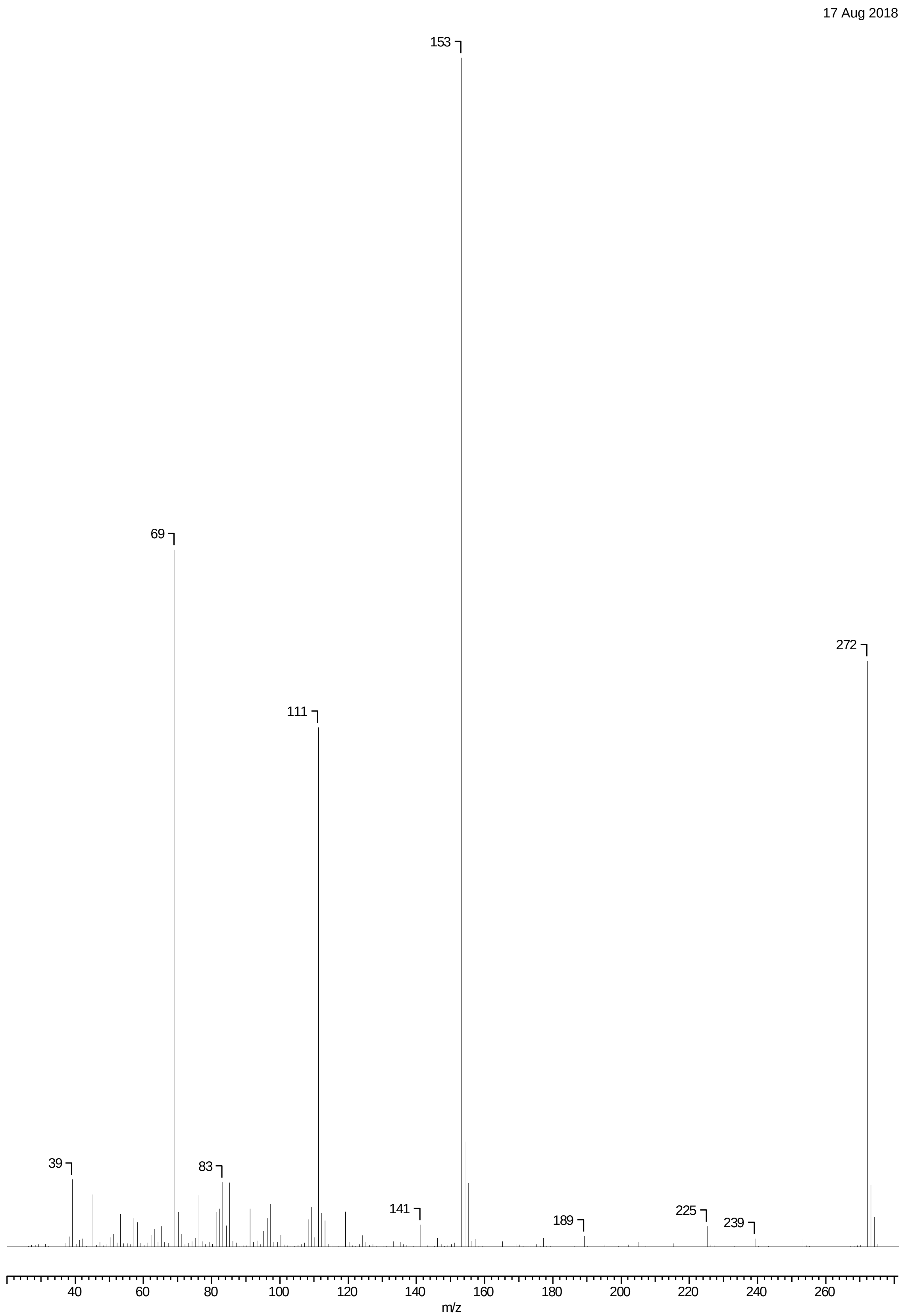

17 Aug 2018
153
69
272
111
39
83
141
189
225
239
40
60
80
100
120
140
160
180
200
220
240
260
m/z

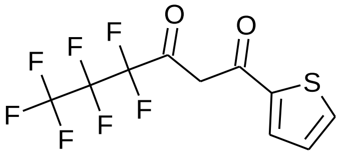
O
O
S
F
F
F
F
F
F
F

3b

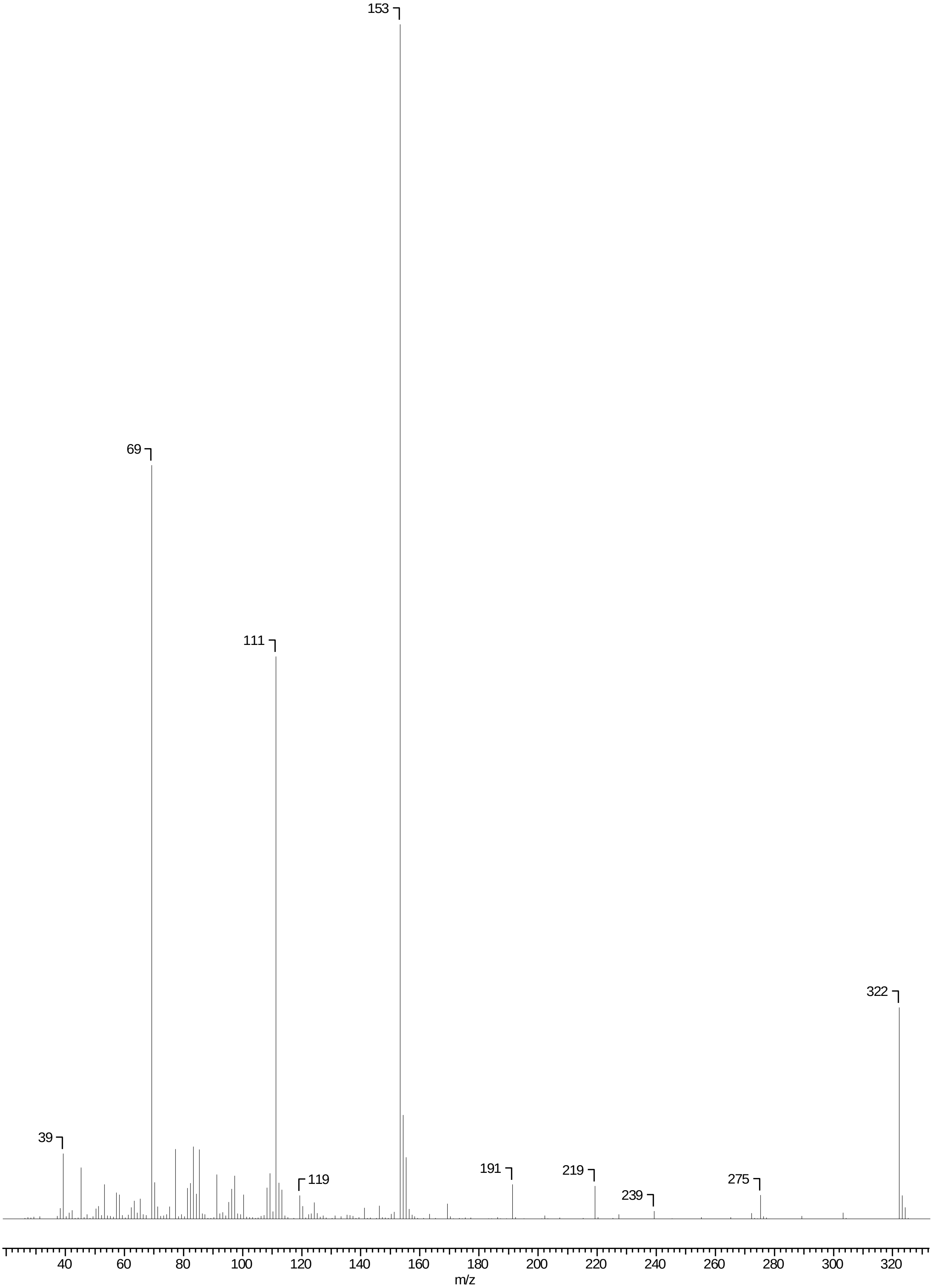
153
69
111
39
119
191
219
239
275
322
40
60
80
100
120
140
160
180
200
220
240
260
280
300
320
m/z

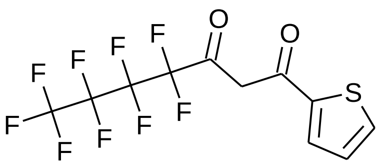

O
O
F
F
F
F
F
F
F
F
F
S

3c

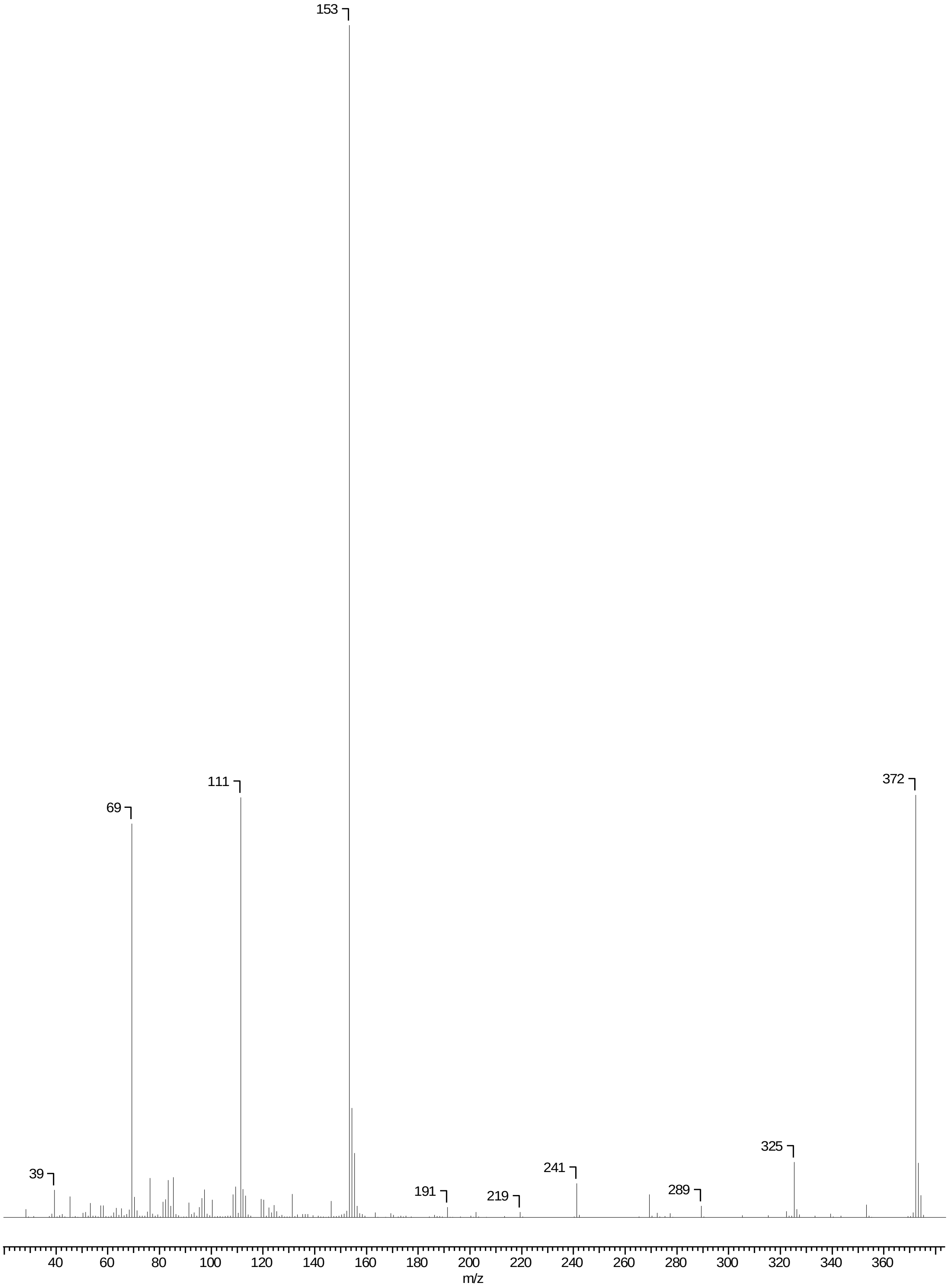

153
111
69
372
39
191
219
241
289
325
40
60
80
100
120
140
160
180
200
220
240
260
280
300
320
340
360
m/z

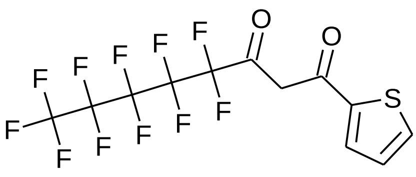
F
F
F
F
F
F
F
F
F
F
F
F
F
O
O
S

3d

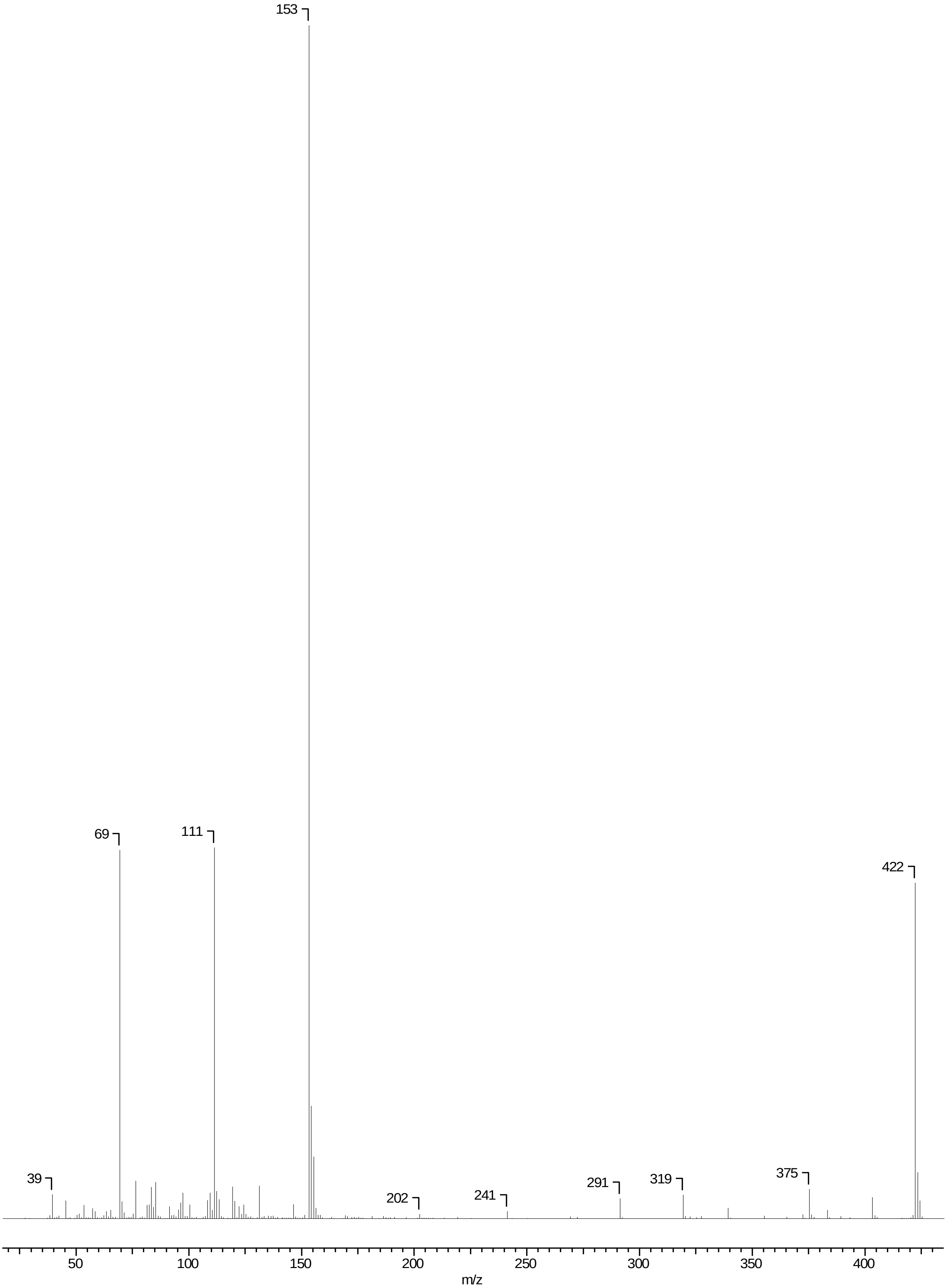
153
69
111
422
39
202
241
291
319
375
50
100
150
200
250
300
350
400
m/z

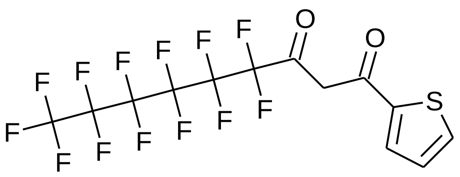
O
O
S
F

3e

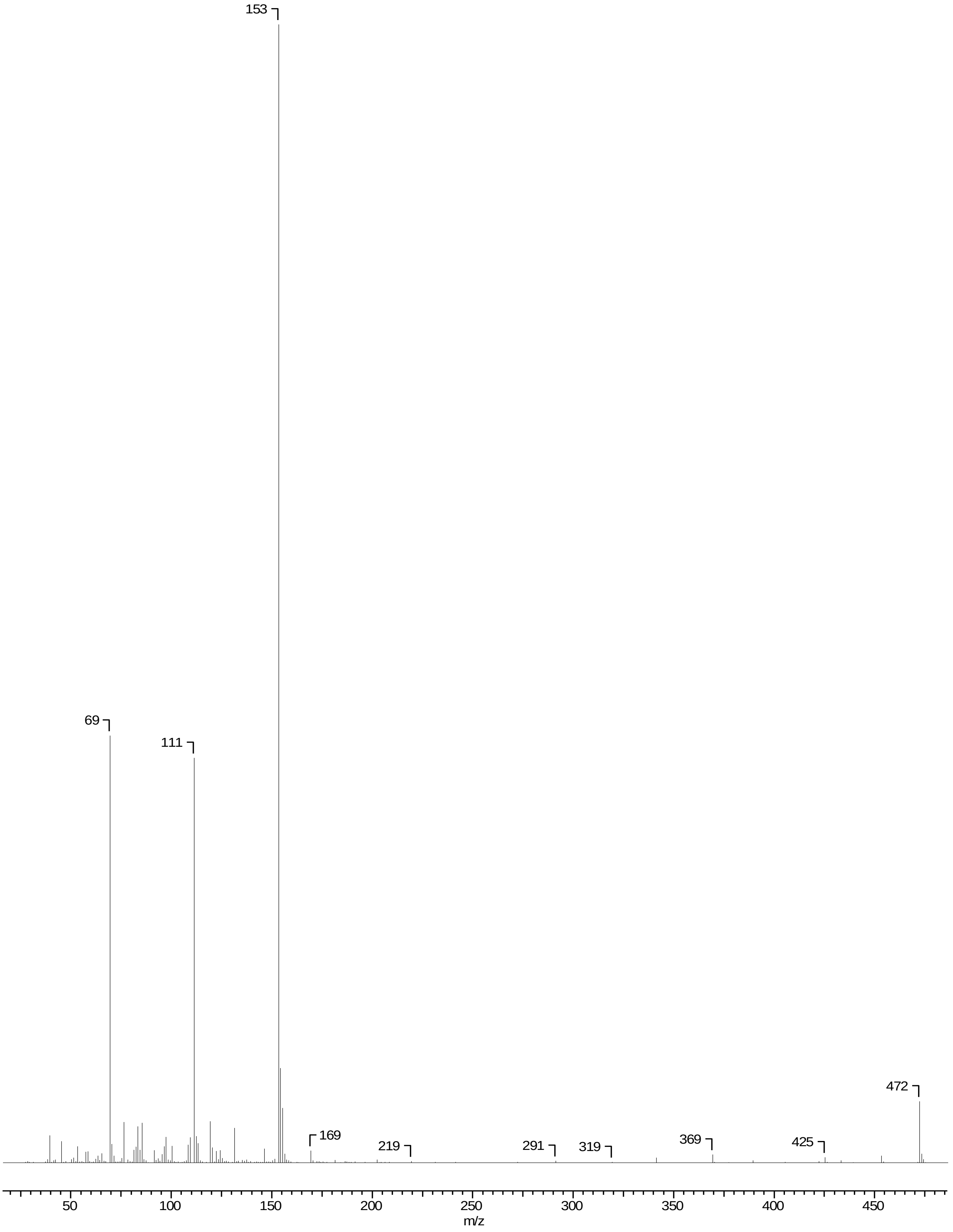
153
69
111
169
219
291
319
369
425
472
50
100
150
200
250
300
350
400
450
m/z

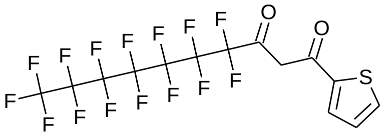

3f

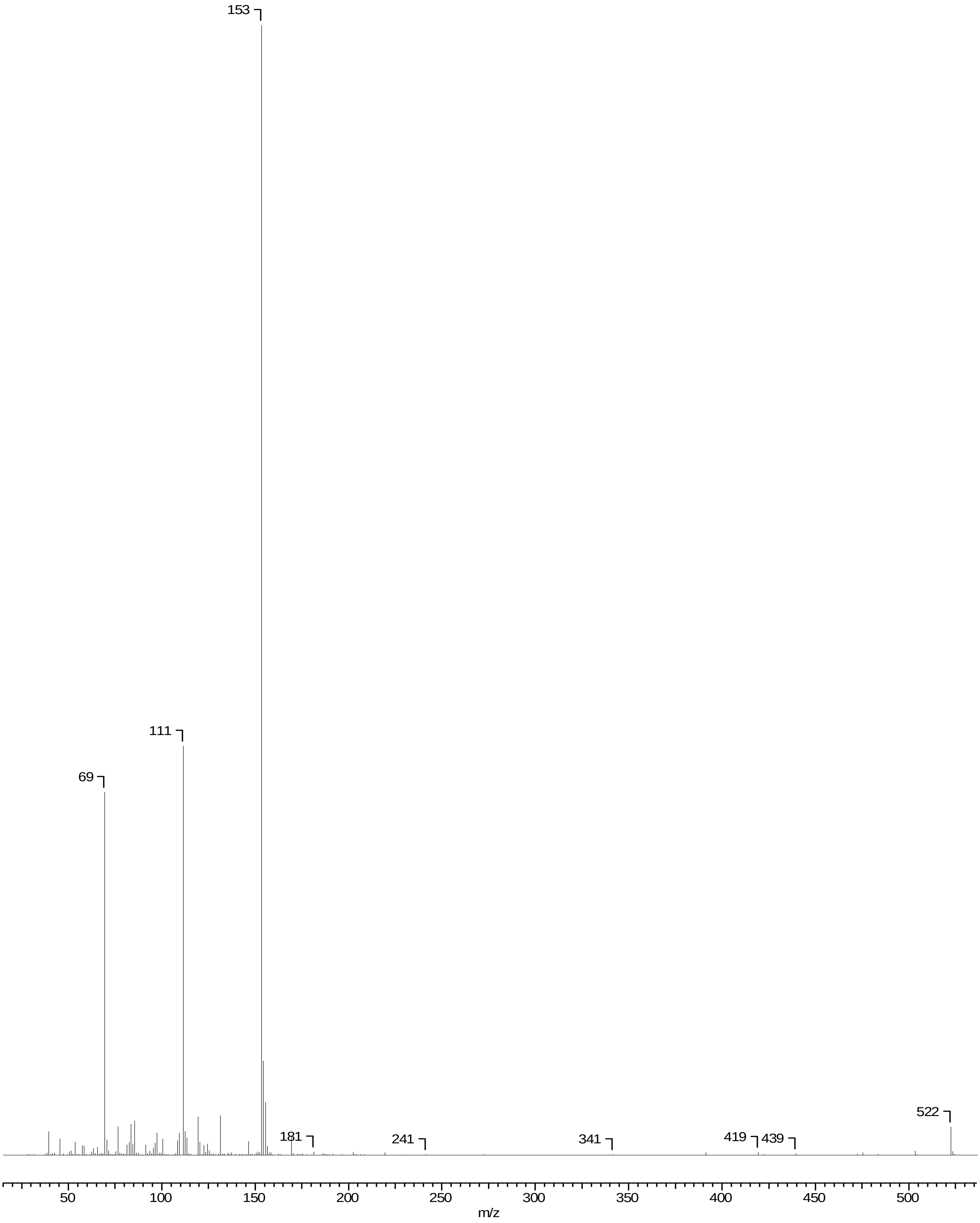
153
111
69
181
241
341
419
439
522
50
100
150
200
250
300
350
400
450
500
m/z

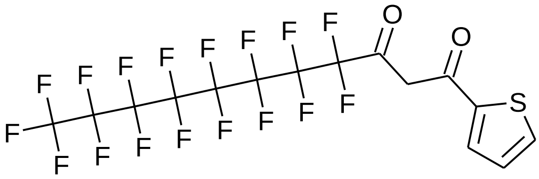
F
O
S

3g

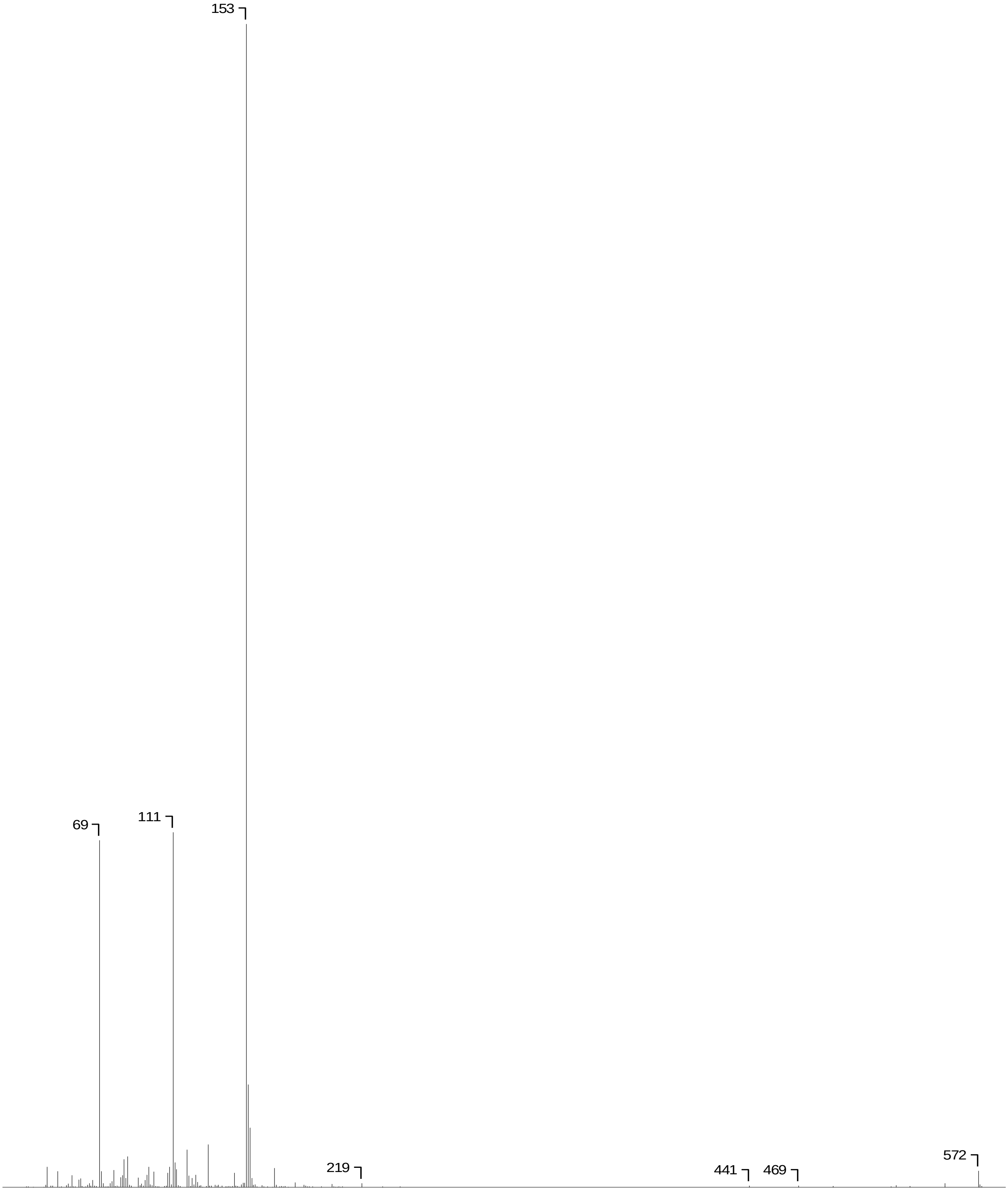
153
69
111
219
441
469
572
50
100
150
200
250
300
350
400
450
500
550
m/z

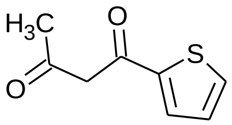
H3C
O
O
S

5

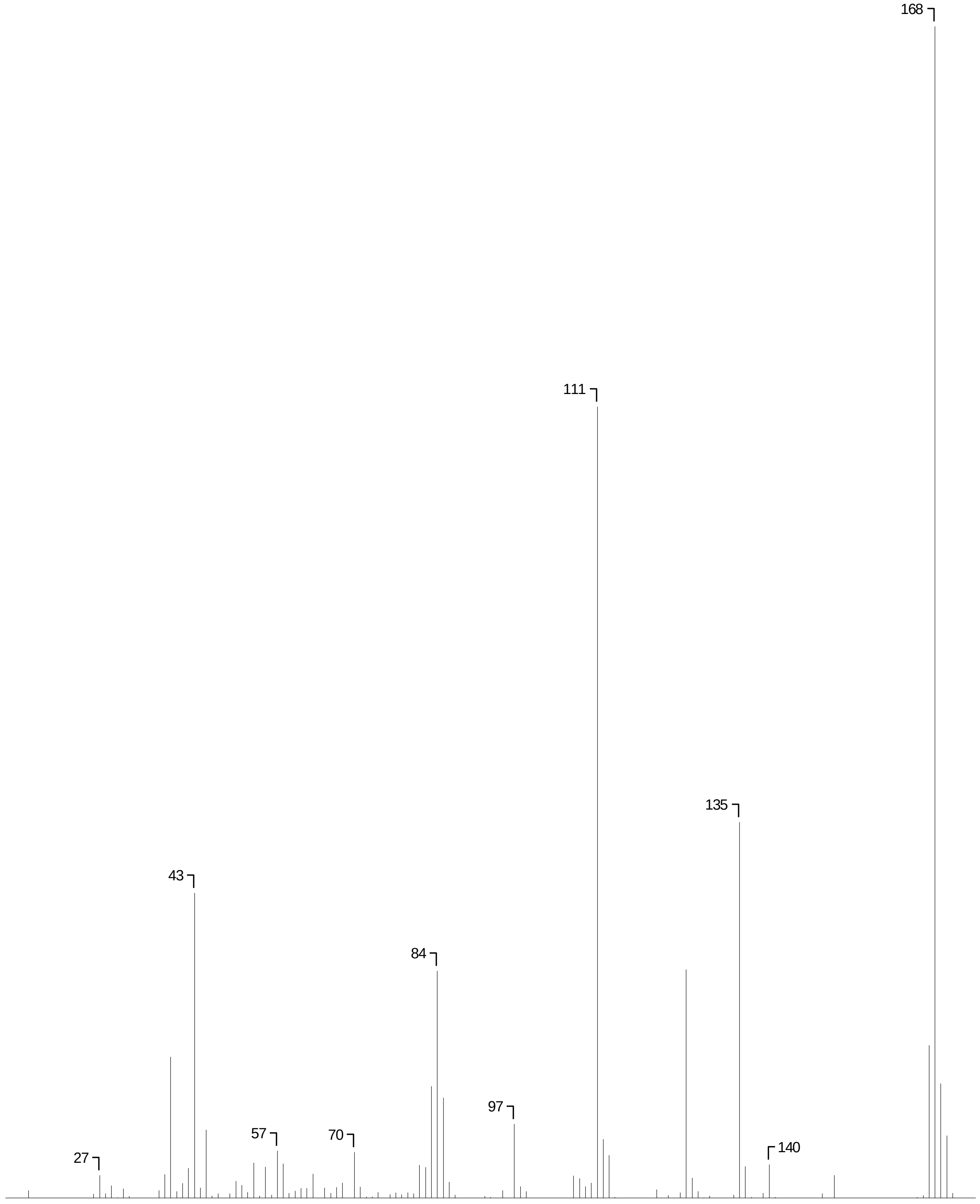
168
111
135
43
84
97
57
70
27
140
20
30
40
50
60
70
80
90
100
110
120
130
140
150
160
170
m/z
